# Polymer Microfiltration and Ultrafiltration Membranes Containing Inorganic Modifiers: Functional Properties and Performance—Review

**DOI:** 10.3390/polym18182304

**Published:** 2026-09-20

**Authors:** Yuliya S. Dzyazko, Valentina V. Chmilenko, Priscila Pini Pereira, Ludmila M. Rozhdestvenska, Katerina O. Kudelko, Nicole Novelli do Nascimento, Angélica Marquetotti Salcedo Vieira, Letícia Nishi, Rosângela Bergamasco

**Affiliations:** 1 V.I. Vernadskii Institute of General and Inorganic Chemistry, National Academy of Science of Ukraine, Palladin Ave. 32/34, 03142 Kyiv, Ukraine; vali4ka.v.ch1998@gmail.com (V.V.C.);; 2 Department of Chemical Engineering, State University of Maringá, 5790 Colombo Ave., Maringá 87020-900, PR, Brazil; 3 Department of Food Engineering, Faculty of Animal Science and Food Engineering, University of São Paulo, R. Duque de Caxias, 225—Jardim Elite, Pirassununga, São Paulo 13635-900, SP, Brazil; 4 Department of Food Engineering, State University of Maringá, 5790 Colombo Ave., Maringá 87020-900, PR, Brazil; amsvieira@uem.br

**Keywords:** antifouling resistance, inorganic modifiers, nanocomposite membranes, microfiltration, ultrafiltration, 3D printing

## Abstract

Modifying MF and UF polymer membranes with hydrophilic compounds is one of the well-known antifouling strategies, which also allows one to achieve a compromise between fluid permeability and the retention of one or another species. Hydrophilizing inorganic substances are widely used for modifying due to their enhanced chemical stability and multifunctionality. In this review, the inorganic modifiers are categorized according to their effect on organic contaminants and bacteria: (i) non-destructive (natural and synthetic aluminosilicates, phosphates and oxides of multivalent elements, particularly magnetic nanoparticles), (ii) bacteria-destructing (silver nanoparticles, advanced carbon nanomaterials, MXenes), and (iii) strongly destructive, which disrupt both bacteria and organic substances (photocatalysts). The mechanism of antibacterial activity of different modifiers is considered. The main approaches to modifying are stressed: blending polymers with preliminarily formed nanoparticles followed by membrane formation, synthesis of them inside the pores of membranes or on their surface, embedding pre-formed particles into pores, or modifying the surface with them. A comparative analysis of the functional properties and performance of pure polymer membranes and materials containing inorganic modifiers has been conducted. The attention is focused on the ability of modified membranes based on various polymers to retain both macromolecular and low-molecular-weight substances together with high fluid permeability and stability against organic contaminants and biofouling. The limitations of materials containing various types of inorganic modifiers are analyzed. Among the possible directions of further investigations, the development of a universal theoretical approach to modifying is noted; moreover, the testing of the materials has to involve the treatment of real solutions.

## 1. Introduction

Membrane technologies are the most dynamically developing separation methods in the world. According to the forecast, the global market for filtration membranes (mainly made of polymers) will exceed 60 billion USD in 2030 [1]. Among filtration materials, half of the market is occupied by porous microfiltration (MF) and ultrafiltration (UF) membranes due to their wide application field.

MF membranes retain relatively large species (from 100 nm up to several microns) due to the sieving effect [2,3,4], they are used for oil–water separation [5], the pretreatment of seawater prior to reverse osmosis (RO) [6], wastewater pretreatment before other purification methods [7], in the food and beverage industry [8,9], particularly for drink disinfection [8], and for other purposes. The separation ability of UF membranes is caused by sieving, electric and dielectric exclusion [4]. They are employed for the separation of smaller species (3–100 nm, another range is 10–100 nm) [2,3,4,5,10,11]. The application field of UF materials involves water [11,12] and wastewater [11,13] treatment, the food [14,15] and pharmaceutical [16] industries, and biotechnologies [17]. Besides the retention of colloidal particles and macromolecular substances, UF membranes are able to concentrate low-molecular-weight compounds: dyes [18], pesticides [19], pharmaceutically active compounds (PAC) [20], and bioactive substances, for instance, polyphenols [21]. Polyelectrolyte-assisted UF [22] or a combination of UF with coagulation [23,24] and coagulation–flocculation [24] allows one to remove heavy metal ions [22,23] or dyes [24] from aqueous solutions. Both UF and MF membranes are used in membrane bioreactors (MBR) [25,26]. This MBR technique combines a biological method for wastewater treatment followed by membrane separation.

Due to the practical importance of polymer MF and UF membranes, a number of investigations are directed to the improvement of their functional properties, such as fluid permeability, the ability to retain one or another species, mechanical durability, and antifouling resistance. Especially the stability against fouling is in the focus of attention, since it impairs the separation efficiency during filtration, deteriorating the quality of a target product (concentrate or permeate). The intensity of membrane contamination depends not only on the operating conditions and composition of the solution, but also on the membrane material. The contamination problem is especially important for hydrophobic polymers, such as polysulfone (PS), polyethersulfone (PES), polyvinylidene fluoride (PVDF), polypropylene (PPP), expanded polytetrafluoroethylene (ePTFE), polyvinyl chloride (PVC) etc. These polymers are traditionally applied to the manufacture of MF and UF membranes due to their thermal, chemical and mechanical stability [4,27]. Normally the water contact angle (WCA) exceeds 90° for these materials.

A common approach, which allows one not only to reduce fouling but also to improve other functional properties of polymer membranes, is modifying polymers with various additives. For instance, hydrophilic organic fragments are grafted to the membrane surface [28]. Another approach is to coat the surface with hydrophilic polymers or to blend hydrophobic and hydrophilic polymers prior to membrane formation. However, grafting is accompanied by the oxidation of polymers, the surface films can be easily damaged, and the blended polymers show poor compatibility. Inorganic modifiers in certain amounts are characterized by better performance. They are more chemically stable than hydrophilic polymers, and many of them do not swell in water, providing the integrity of polymer–inorganic composites. Inorganic modifiers also possess thermal stability; this property is important for 3D printing. Moreover, they are multifunctional. For instance, Ag nanoparticles (AgNPs) not only ensure the antibacterial activity of PVDF-based membranes but also improve their mechanical properties and enhance hydrophilicity [29]. Similarly, polymer membranes modified with TiO_2_ are characterized by not only photocatalytic activity, but also higher mechanical strength and more considerable hydrophilicity than pristine polymers [30]. Despite a large number of studies, the information about polymer membranes modified with various inorganic additives is fragmented and sometimes even contradictory. Previous works are focused mainly on one or another type of modifier, for example, AgNPs [31], photocatalysts [32], MXenes [33] and advanced carbon nanomaterials [34,35]. Other directions are modifying membranes based on one or another polymer, such as PES [36], PVDF [37], PS [38], and PVC [39]; both organic and inorganic modifiers are described. Modified membranes are also considered in the context of their application in a particular field, for instance, in the food industry [40], MBR [41], and the pulp and paper industry [42]. Modifying is often discussed together with other antifouling strategies. Here the focus of our attention is the effect of nanoparticles of different inorganic compounds on the functional properties and performance of MF and UF membranes produced from a wide range of polymers.

Although modifying nanofiltration (NF) [43] and RO [44] membranes with inorganic nanoparticles is described in the literature, this review is focused specifically on MF and UF membranes, since the modifier effect on their functional properties is the most prominent. For instance, the membranes produced from PS show no salt retention, but the addition of graphene oxide (GO) to the polymer allows one to obtain materials which retain Pb(NO_3_)_2_ [45]. Moreover, modifiers can minimize the fouling of MF and UF membranes, which is caused by organic substances and microorganisms. At the same time, inorganic salts make a great contribution to the fouling of NF and RO membranes (this type of fouling is called scaling). The modifiers are not effective against scaling.

In this review, the aim and tasks of membrane modifying are formulated. Based on the analysis of representative publications, it is stressed that one of the important objectives is to provide the antifouling resistance of polymer membranes. For the first time, inorganic modifiers are categorized according to their effect on organic contaminants and bacteria, and the main approaches to modifying polymer membranes are presented. The paper gives comparative information about the functional properties and performance of pure polymer membranes and materials containing inorganic modifiers. The attention is focused on the ability of modified membranes to ensure high retention of both macromolecular and low-molecular-weight substances together with high fluid permeability and stability against organic contaminants and biofouling. Additionally, the mechanism of antibacterial activity of various inorganic modifiers is considered. The information about 3D-printed polymer membranes containing inorganic constituents is discussed separately, since modified materials, which are obtained in this manner, are still little studied.

Nanofiltration (NF) and RO membranes are outside this review, since inorganic compounds make a great contribution to their fouling together with organic substances. Due to the sieving effect, colloids and macromolecular substances are deposited on the outer surface of these membranes, from which they can be easily removed. However, these substances are accumulated in the pores of MF and UF membranes. Here we consider inorganic nanoparticles, first of all, as protectors against pore fouling. Nevertheless, various inorganic modifiers are used to reduce the contamination of NF and RO membranes with microorganisms and also with low-molecular-weight organic substances [43,44].

## 2. Modifying with Inorganic Nanomaterials as a Strategy to Improve the Membrane Performance

### 2.1. Aims and Tasks of Modifying

The purposes of modifying are to improve the performance of filtration membranes and to extend their lifetime. Modifying performs the following tasks [46]:

- Achieving a compromise between the retention of one or another substance and the filtration rate. Normally the retention increases with pore narrowing; simultaneously molecular weight cut-off (MWCO) decreases. However, pore narrowing depresses the permeate flux;
- Improving the porous structure of membranes by minimizing the tortuosity, narrowing–widening and branching of pores, since the fastest fluid transport is achieved in straight, uniform voids;
- Enhancing the mechanical properties of membranes;
- Changing the morphology and chemical composition of the membrane surface, which acquires new properties. For instance, magnetic nanoparticles grafted via polymer spacers reduce concentration polarization [47];
- Providing antifouling resistance to membranes, since fouling significantly slows down filtration, deteriorates the quality of a target product, and increases energy consumption, because higher pressure is necessary. Membrane cleaning requires a considerable volume of reagents for regeneration and pure water for rinsing. Chemical treatment reduces the membrane lifetime. Thus, investigations of modified membranes always involve testing their stability against fouling. Due to the importance of this problem, we will further briefly consider the types of membrane fouling and antifouling strategies.

### 2.2. Fouling. Types of Fouling, Its Quantitative Estimation and Antifouling Strategies

Membrane contamination is caused by physical and chemical interactions between different fouling components in the solution, as well as by interactions between these components and the membrane. The fouling intensity is determined by the solution composition (pH, ionic strength and concentration of components, their molecular weight, and the shape and size of particles), membrane properties (morphology of the surface, its charge, hydrophobicity, pore size), and operating conditions (temperature, degree of solution concentrating, hydrodynamic conditions) [48]. According to their nature, the fouling of MF/UF membranes can be classified into four main types [49] (typical morphology of fouling is shown in Figure 1A–D [50]):

- Insoluble inorganic or organic particles;
- Organic dissolved components and colloids (for instance, humic and fulvic acids, proteins);
- Inorganic dissolved components (iron, manganese, silica compounds), which are deposited due to oxidation or changes in pH. Coagulant/flocculant residues can also act as contaminants;
- Microorganisms (algae, bacteria, fungi), which cause biofilm formation (biofouling). The biofilm evolution involves the development of a conditioning layer of exopolysaccharides produced by bacteria, bacteria adsorption, their adhesion, biofilm growth and destruction (Figure 1E [51]).

The shape of inorganic particles is rather regular, the particles of organic substances are irregular, biofouling is expressed as rod-like and spherical particles, colloidal particles form a film consisting of flakes with holes between them (see Figure 1A–D).

UF membranes can be contaminated during the filtration of solutions containing even soluble compounds, such as dyes. Under concentration polarization, their content in the boundary solution at the membrane surface can exceed their solubility [4]. For instance, poorly soluble dyes precipitate, contaminating the membrane [18].

According to cleaning methods, fouling can be classified into reversible and irreversible [49,52]. The difference between them depends entirely on the operating and cleaning conditions. Reversible fouling is caused by a layer of sediment on the membrane surface. A membrane with reversible fouling can be regenerated by an appropriate physical washing protocol, which includes backwashing or hydrodynamic cleaning (surface washing with hydrodynamic pulsations). Irreversible fouling is due to chemisorption, mainly in pores. It is formed even in the absence of filtration due to the hydrophobic interactions of the foulant with the polymer, hydrogen bonding and van der Waals attractions. This fouling can be removed by chemical cleaning; in some cases, it is necessary to replace the membranes.

The fouling effect on the permeate flux is quantitatively expressed in terms of the flux decline ratio (*FDR*) and flux recovery ratio (*FRR*). These parameters are used by many authors, for instance, in [53]. The expressions for the calculation of these parameters are:

(1) $$FDR=\left(1-\frac{J_{2}}{J_{1}}\right)\times 100\%;$$

(2) $$FRR=\frac{J_{3}}{J_{1}}\times 100\%.$$

Here *J*_1_ and *J*_3_ are the pure water fluxes through the membrane before and after the solution filtration respectively, and *J*_2_ is the permeate flux during the solution filtration. The higher the *FRR* parameter and the lower the *FDR* parameter, the higher the antifouling resistance. Pure water permeability (PWP, i.e., water flux related to pressure) is a functional property of membranes. The contributions of reversible (*R_r_*) and irreversible (*R_ir_*) fouling to the *FDR* parameter are calculated as:

(3) $$R_{r}=FRR-(100\%-FDR);$$

(4) $$R_{ir}=100\%-FRR.$$

Particles whose size is close to that of the pores block them [48,49,52]. Smaller particles pass through the membrane or are adsorbed on the pore walls, making them narrower. Larger particles precipitate on the outer surface of the membrane. Convection, Brownian diffusion, hydrodynamically induced diffusion, particle interaction in the fluid, and gravitational deposition are the main mechanisms of particle transport to the membrane surface [54].

As a rule, the first stage of fouling is the filling of pores, and then the precipitate layer (the so-called “cake”) is formed. This layer, which is compacted under pressure, acts as an additional membrane, since it has numerous irregular pores of different sizes [55]. Thus, the cake controls the particle retention and permeate transport.

Thus, according to the location of the particles and their effect on the porous structure of membranes, fouling is categorized as follows [48,49,52]:

- Pore blocking;
- Pore narrowing;
- Cake formation.

An intermediate mechanism (between pore blocking and cake formation), according to which the particles deposit on already blocked pores, is also noted [56]. One or another mechanism dominates depending on the particle size, operating conditions and membrane material.

Strategies to reduce fouling involve the choice of a membrane with a certain MWCO, since its pores minimize the penetration of one or another foulant (depression of irreversible fouling). Other ways are adjusting the pH and ionic strength of the solution, controlling its temperature, minimizing concentration polarization by reducing pressure and increasing the rate of flow along the membrane (for tangential modules), and solution pre-treatment by coagulation, adsorption, filtration through filters with larger pores, etc. [49,55]. These strategies allow one to reduce both reversible and irreversible fouling.

A widespread approach is to make the membranes resistant to fouling by means of modifying them with various substances. Depending on the location of the modifier particles, they provide the formation of a hydration layer on the outer surface and/or in the pores during the initial contact of the membrane with water. This hydration layer depresses the adsorption of organic substances and particularly minimizes the formation of a conditioning layer, reducing biofouling. Besides decreasing total fouling, modifying allows one to diminish the contribution of irreversible fouling.

Various approaches to modifying membranes, as well as inorganic nanomaterials, which are used for this purpose, are discussed below.

### 2.3. Main Types of Inorganic Modifiers

The advantages of inorganic modifiers over hydrophilic polymers [28], enzymes [57], biocidal compounds [58], metal–organic [59] and covalent–organic frameworks [60], which are also applied to membrane modification, are the following: a wider field of action (besides hydrophilization, many inorganic modifiers can destroy bacteria and even organic substances), higher chemical stability (especially against oxidizing agents), the improvement of mechanical properties (when the polymer contains a small amount of the modifier) and thermal stability (important for 3D printing). Many modifiers do not swell in water, providing good compatibility with polymers. A number of them can be obtained from relatively cheap (and even low-cost) substances by means of simple synthesis procedures; some inorganic modifiers (carbon nanodots, CDs or zeolites) are synthesized via a “green” hydrothermal method.

All inorganic modifiers hydrophilize polymers, which are applied to the manufacture of MF and UF membranes (originally, these polymers are more or less hydrophobic). The hydrophilicity of reduced graphene (rGO) is controlled during GO reduction. Hydrophobic carbon nanotubes, fullerenes and nano-onions are usually functionalized prior to membrane modification. Graphitic carbon nitride (g-C_3_N_4_) is also hydrophobic, but it is used together with photocatalysts, which hydrophilize polymers. As a result of hydrophilization, the polymers become more stable against fouling and biofouling.

According to their action, modifiers can be categorized into three types: non-destructive (synthetic inorganic ion exchangers, clay minerals, magnetic nanoparticles), destructive (advanced carbon nanomaterials, MXenes, AgNPs) and strongly destructive (photocatalysts), as seen in Figure 2. The materials of the first type leave both bacteria and organic substances. The modifiers that belong to the second type cause bacterial degradation but leave organic substances intact. At the same time, the nanoparticles of photocatalysts can destroy both bacteria and organic compounds. Photocatalysts can be applied to filtration and membrane cleaning [61].

Modifying with inorganic substances sometimes involves self-polymerized organic compounds, for instance, dopamine [62] or tannic acid [63]. Their functions are to stabilize the modifier layer, enhance the retention ability of the membrane, and provide its additional hydrophilization. The modifying procedures are considered further.

### 2.4. Modifying Procedures

The modifying procedures can be divided into three main types (Figure 3):

- The first type involves blending previously formed nanoparticles with a polymer solution followed by membrane formation. Subsequently, conventional methods, for instance, phase inversion or electrospinning, are employed for membrane preparation. This approach is applied to all modifiers, especially to natural nanomaterials or substances whose manufacture requires harsh conditions (photocatalysts, MXenes, advanced carbon nanomaterials, functionalized nanomaterials, etc.). It is possible to obtain membranes with more or less uniformly distributed particles in polymers. As a result, the nanoparticles are located not only in the bulk of the polymer but also on the pore walls and at the outer surface of the resulting membrane. Embedding nanoparticles is the most widely used approach since it provides the fixation of nanoparticles in polymer matrices. However, the limitation of blending is the poor dispersibility of carbon-free inorganic modifiers in organic solvents;
- The second type involves the synthesis of the modifier directly within the pores of pre-formed membranes or on their outer surface (AgNPs, phosphates or hydrated oxides of multivalent metals);
- The third type involves modifying membranes with pre-formed nanomaterials by means of surface engineering. For instance, magnetic [47], and TiO_2_ nanoparticles [64], as well as AgNPs [65] are grafted to the surface. The modifier can also be embedded into the pores [66,67,68].

A combination of different approaches to modifying is possible. TiO_2_, which was contained in the polymer, was subsequently doped with Au [69]. GO [67] or CDs [70] were mixed with a solution of zirconium hydroxocomplexes, and then carbon nanomaterials precipitated together with hydrated zirconium dioxide inside the pores [68,69]. Thus, the modifying methods involve both the second and third approaches.

The problems of the first approach are to achieve a homogeneous distribution of embedded nanoparticles in the polymer, avoid their aggregation, and the delamination of the resulting composites. The second and third approaches can be applied to both experimental samples and commercial membranes. The modifying conditions (chemical composition of solutions, thermal treatment) must ensure no polymer degradation. Nevertheless, polyacrylonitrile (PAN) and polyamide (PAM) membranes were modified with hydrated zirconium dioxide in acidic and alkaline media, where these polymers undergo hydrolysis [19,68]. Specifically, the hydrolyzed polymers perform the function of a binder, reducing the leakage of nanoparticles under pressure. Hydrolysis improves the retention ability of UF PAN membranes themselves evidently due to a decrease in pore size (MWCO decreases from 20 down to 6 kDa) [71].

The problem with filling pores is to place the modifier particles specifically in the active layer. When aqueous solutions are used for modifying, the hydrophobic polymer has to be preliminarily hydrophilized to provide access for the reagents to the pores. Surface engineering often requires preliminary activation of the membrane surface. This is not always achievable, for instance, when the inner surface of a tubular membrane has to be activated with irradiation. The formation of a modifier layer on this surface is also problematic. Additionally, surface activation can deteriorate the mechanical properties of polymers.

Inorganic modifiers strongly influence the morphology, functional properties and performance of the resulting membranes. The effects of inorganic nanoparticles are considered further.

## 3. Effect of Inorganic Nanoparticles on Morphology and Functional Properties of Modified Membranes

### 3.1. Morphology of Modified Membranes

The pore shape and size of filtration membranes are among the key factors determining the efficiency of membrane separation. As a rule, the pores of MF and UF materials are observed with scanning electron microscopy (SEM) [4]. Mesopores, which are responsible for the separation ability of UF membranes, are identified by the method of nitrogen adsorption–desorption. Macropores can also be determined with the mercury intrusion technique. However, this method gives an error in the region of small pores due to their degradation under high pressure. Other methods, particularly standard contact porosimetry, which is categorized as a non-destructive technique, are described in [72]. Sharp peaks of differential pore-size distributions correspond to straight pores without narrowing–widening; specifically, these pores provide high fluid transport.

The ambiguous effect of modifiers on the pore structure of filtration membranes is noted in many papers. For instance, no effect of MXene on the size of pores in the polysulfate active layer is reported (Figure 4a) [73]. However, this layer becomes looser. CD additions to PES increase the pore size; however, it is independent of the modifier content [74]. Alternately, a small amount of MXene (up to 0.2 wt%) decreases the pore size of PES; it reaches 33 nm at 0.5 wt%, and further decreases down to 20 nm [75]. An increase in the zeolite amount depresses porosity and makes the active layer thicker [76]. Various effects of modifiers on the shape of the membrane pores should be stressed. For instance, fullerenes [77] and nano-onions [78] straighten the pores of polyethylene oxide and PES respectively, while functionalized GO makes them more tortuous but larger [79] (Figure 4b–f).

The effect of modifiers on the porous structure of polymers is due to their cross-linking ability. Interfacial bonding between the particles and the polymer via surface groups is suggested for MXene embedded into PVDF [80]. Modifiers such as carbon nano-onions [78], fullerenes [77], and CNTs (Figure 5a) [81] can also be coupled with the hydrophobic regions of polymers. Cross-linking causes more ordered packing of polymer chains, which reduces their topological entanglements. The interaction depends on the types of modifier and polymer. The modifier amount also plays a significant role, since it determines particle aggregation. The aggregates are unevenly distributed in the polymer, as seen in the example of AgNPs (Figure 5b–e) [82]. The size of inorganic particles, their aggregation, and their location (concentration at the surface, or homogeneous distribution in the polymer) result in varying surface roughness, which is determined by atomic force microscopy (AFM). The effect of the modifier content on the roughness is also ambiguous (see Figure 4a). The roughness affects the formation of reversible fouling and its removal from the surface.

Thus, the amount of modifier, the state of nanoparticles (aggregated or non-aggregated), their distribution in the polymer, and their interaction with the polymer chains affect the size and shape of pores, as well as the thickness of the active layer. These factors influence fluid permeability (particularly PWP) and the ability of modified membranes to retain one or another species. The separation ability of UF membranes is estimated via calibration with standard macromolecular compounds: bovine serum albumin (BSA), ovalbumin (OVA), polyethylene glycols, polyvinylpyrrolidones or dextrans of different molecular mass [4]. The effect of various inorganic modifiers on the retention of these species is described further.

### 3.2. Hydrophilicity and Surface Charge

Due to their hydrophilizing effect, inorganic modifiers allow one to decrease the adsorption of organic species on the membrane surface. Thus, it is possible to reduce fouling with organic substances and also biofouling, since the formation of a conditioning layer is depressed. The effect of a modifier on membrane stability against fouling is preliminarily determined by measuring the WCA using water as a working liquid [83]. The membrane surface is hydrophilic when the WCA is less than 90°. If it exceeds 90°, the surface is hydrophobic. The WCA value strongly depends not only on the chemical composition of the surface but also on its roughness, membrane porosity and pore size. When a membrane is highly porous, the WCA can be small despite the low hydrophilicity of the material. Conversely, a rough surface shows a higher WCA than a smooth one when the membrane material is similar. As a rule, membranes with low WCA demonstrate high PWP. In the case of solutions, the initial contact with the membrane leads to the formation of a hydrated layer on the surface, reducing further adsorption of organic substances. The methods of immersion, sessile drop, sticking bubbles, and captive bubbles are applied to WCA measurements. However, membranes coated with GO can show a higher WCA than pristine polymers. At the same time, high PWP has been found. This contradiction is considered in Section 6.1.2.

When inorganic nanoparticles are distributed in the polymer or embedded in the membrane pores, they reduce irreversible fouling due to a sieving and/or hydrophilizing effect. Another result is an enhancement of fluid permeability. To determine hydrophilic and hydrophobic porosity, the Archimedes (pycnometer) method can be employed. This technique involves ideally wetting working liquid (octane) and water [72]. The “octane” porosity corresponds to all pores; the porosity determined with water is attributed to hydrophilic pores. Hydrophobic porosity is found as the difference between the values of total and hydrophilic porosity. Modifying PAN and PAM membranes with an inorganic ion exchanger increases hydrophilic porosity and reduces the volume of hydrophobic pores [19]. Standard contact porosimetry allows one to obtain pore-size distributions both in aqueous and non-aqueous media [72].

The antifouling resistance of the membranes strongly depends on their surface charge, since a more highly charged surface shows stronger hydrophilicity [83]. To determine this characteristic, the zeta potential is estimated by measurements of streaming potential or electrophoresis. The point of zero charge (PZC) can be determined from the curves of potentiometric titration [84]. This approach is effective when the modifier contains ion-exchange groups.

When inorganic nanoparticles enhance the zeta potential, the membrane is expected to be more stable against fouling than the pristine polymer material. Negatively charged organic or inorganic particles are repelled by the negatively charged surface, whereas positively charged species are attracted to it. Since bacteria are usually negatively charged, they are repelled by the negatively charged membrane. However, the charge of both the membrane and the species strongly depends on the solution pH. Thus, the acidity of the solution must be taken into consideration.

It should be noted that the positive effect on membrane hydrophilicity and surface charge is pronounced when the modifier content is 0.1–0.5% (Figure 6) [73,85,86,87,88,89,90]. Further increasing its amount causes deterioration of these properties [73,85,88] or no further improvement [86,87,89,90]. This is explained by the aggregation of nanoparticles and their uneven distribution. When the pores of a preliminarily formed membrane are filled with nanoparticles, the WCA is lower than that of pristine polymers despite a high modifier content (3–15 wt%) and lower porosity [19,81]. In this case, polymer chains are largely not in contact with the polymer chains, as the particles touch upon the pore walls.

### 3.3. Effect of the Modifier Content on the Membrane Performance

The performance of modified membranes is determined by their porous structure on the one hand, and hydrophilicity on the other hand. In the case of materials obtained by blending polymer and inorganic constituents, increases in PWP, retention of species, and the enhancement of antifouling resistance are reported for membranes with a modifier amount of ≤0.5 wt% (Figure 7) [85,86,87,88,89,90]. Further increasing the content of the inorganic constituent results no improvement of the membrane performance or even its degradation. Nevertheless, membranes with higher modifier content have been investigated; it can reach 3 [91], 7 [92] or even 30% wt% [93], and a positive effect of inorganic constituents has been reported. The performance of the materials that contain modifiers only on their outer surface is very sensitive to the thickness of this layer [94,95].

### 3.4. Mechanical Properties of Modified Membranes

The effect of nanoparticles on the mechanical properties of modified membranes is stressed in a number of papers [29,30,45,79,96,97,98,99,100,101,102,103,104,105,106]. Compared with pristine polymers, modified membranes show higher values of tensile strength, Young’s modulus, and elongation at break (Table 1). This is explained by the transfer of stress to embedded nanoparticles [96,97], and also by interfacial bonding between them and the polymer (cross-linking, see Figure 5). This bonding increases the polymer crystallinity [79]. Indirect confirmation of cross-linking is the synergism of the membrane constituents (MXene and polyvinyl alcohol, PVA) regarding tensile strength and Young’s modulus [96,97]. The positive effect of the nanoparticles is pronounced despite the higher porosity of modified membranes compared with the pristine polymer [45].

However, the positive effect is detected mainly at a low modifier content. For instance, a significant increase in Young’s modulus was found when the MXene content was ≤0.5 wt% [75]. As shown for GO-containing PES membranes, the influence of the modifier amount is ambiguous [45,79,98]. When it exceeds 5% [79] or 15% [45,98], both the tensile strength and Young’s modulus decrease, since static obstacles, an irregular arrangement, and the aggregation of nanoparticles decrease crystallinity [79]. In addition, interfacial voids between embedded particles and the polymer deteriorate the mechanical characteristics of modified membranes [99,100]. This problem can be solved by the functionalization of nanoparticles, since organic fragments fill the voids. Moreover, these fragments minimize the aggregation of embedded nanoparticles, providing their homogeneous distribution in polymers [89]. Compared with pristine polymers, higher hardness [101] and Young’s modulus [45,79,96,98,99,101] mean considerable resistance of the membrane to pressure. Thus, inorganic nanoparticles behave similarly to a rigid polymer in the pores of flexible acetylcellulose [102] reducing the membrane compression during filtration [103].

Polymers show lower elastic strain after modification, especially with an increase in the modifier content, since the nanoparticles reduce the flexibility of the polymer chains [96]. Nevertheless, higher elastic strain has been found for the GO-containing membrane [79].

## 4. Antibacterial Activity of Inorganic Nanoparticles

Besides the hydrophilization of polymers, AgNPs, MXenes, advanced carbon nanomaterials and photocatalysts can destroy bacteria; this property is also characteristic of membranes containing these modifiers. In this section, the mechanism of the antibacterial activity of inorganic nanoparticles is considered.

### 4.1. AgNPs as an Antibacterial Agent

A number of inorganic modifiers reduce biofouling of UF and MF membranes due to their ability to destroy bacteria. Let us consider the mechanism of the antibacterial activity of inorganic modifiers. Regarding AgNPs, they are oxidized by dissolved oxygen, and Ag^+^ ions are released [107]:

4Ag_s_ + O_2aq_ + 4H^+^ → 4Ag_aq_^+^ + 2H_2_O. (5)

Under the influence of light, AgNPs are oxidized, generating reactive oxygen species (ROS) [108,109]:

(6) $${\mathrm{Ag}}_{s}\;\overset{h\nu }{\to }{{\mathrm{Ag}}_{\mathrm{aq}}}^{+}\;+\;\bar{e};$$

(7) $$\bar{e}+O_{2}\to •{O_{2}}^{-}.$$

Further the superoxide radicals form hydrogen peroxide:

(8) $$•{O_{2}}^{-}\;+\;\bar{e}+2H^{+}\to H_{2}O_{2}.$$

Also H_2_O_2_ can appear in water due to its oxidative pretreatment. This reagent interacts with superoxide radicals, producing molecular oxygen:

•O_2_^−^ + H_2_O_2_ → •OH + OH^−^ + O_2_, (9)

and atomic oxygen:

O_2_^−^ + •OH → 1/2O_2_ + OH^−^ (10)

The adsorption of H_2_O_2_ on the surface of AgNPs is accompanied by the following reaction:

H_2_O_2_(aq) → 2•OH, (11)

which is the most realistic from a thermodynamic point of view [110]. Among other noble metals, specifically the surface of the silver is characterized by the highest adsorption energy for •OH radicals, facilitating the H_2_O_2_ splitting.

The ROS formation reactions are very sensitive to pH. For instance, mechanism (5) is realized in acidic media, whereas H_2_O_2_ splitting becomes more intensive with increasing pH [107]. Moreover, the •OH →•O_2_^−^ transformation in alkaline solutions is suggested:

•OH + H_2_O_2_ → •O_2_^−^ + H_2_O + H^+^ (12)

The degradation of AgNPs is slower in alkaline solutions than in neutral media due to passivation with AgOH. This unstable compound decomposes as follows:

2AgOH → Ag_2_O + H_2_O. (13)

ROS, light and increasing pH also promote the reorganization of AgNPs [111]:

Ag_aq_^+^ + •O_2_^−^ → Ag_s_ + O_2_; (14)

Ag_s_ + •O_2_^−^→•Ag_s_^−^+ O_2_; (15)

•Ag_s_^−^ + Ag_aq_^+^ → Ag_s_ + Ag_s_. (16)

Under the influence of reducing agents and light, the following reaction occurs:

(17) $${{\mathrm{Ag}}_{\mathrm{aq}}}^{+}\;+\;\bar{e}\to {\mathrm{Ag}}_{s}.$$

This means that light induces both Ag oxidation (6) and Ag^+^ reduction (17), an additional reagent (particularly provided by biological media) is needed in the latter case. Reorganization, which is faster for smaller particles, causes a decrease in particle size and a change in their shape. Other pathways of AgNPs degradation and passivation are shown in Figure 8 [112]: AgNPs are sensitive to chlorides, sulfides, thiols and ammonia. All these factors are important for AgNPs located inside the membrane or on its surface. The effect of metal cations is expressed in colloidal solutions since they displace stabilizing ligands and provoke the aggregation of nanoparticles.

Thus, AgNPs produce both Ag^+^ ions and ROS, which are active against bacteria. Moreover, positively charged or uncharged nanoparticles are themselves active since they attach to the negatively charged cell membrane, disturbing its integrity and permeability [44,112]. Compared with larger AgNPs, smaller nanoparticles are more effective due to better contact with microorganisms and a faster release of Ag^+^ ions. After damaging the cell membrane, AgNPs enter the bacteria, intercepting the cellular processes (Figure 9a). The nanoparticles are easily oxidized inside the cell, releasing Ag^+^ ions.

Ag^+^ ions bind to the thiol groups of cysteine and methionine, and to the amino groups of all amino acids [112]. This leads to the destruction of the cell membrane, turning DNA molecules into a condensed form, which possesses no replication ability [44]. Ag^+^ ions also interact with ribosomes, inhibiting the formation of proteins and enzymes. Cl^−^ anions, which maintain the salt balance inside the cell, interact with Ag^+^ cations. The damaged cell membrane cannot retain the cellular contents, particularly respiratory enzymes. They inhibit ROS produced by mitochondria as by-products of adenosine triphosphate synthesis. Mitochondrial disfunction also increases the content of free radicals, provoking oxidative stress. In undamaged cells, ROS are intracellular signaling mediators. However, oxidative stress disables the signal transmission pathways.

ROS produced by AgNPs can destroy both extracellular (lipids and proteins of the peptidoglycan matrix, which is attached to proteins and surrounds the cell) and intracellular (enzymes, coenzymes, nucleic acids) cell components. ROS attack the unsaturated fatty acids of phospholipids, disrupting membrane integrity. Free radicals also oxidize the membrane proteins, breaking the functionality of ion channels, enzymes, and receptors, and, as a result, disrupting the transport of substances and signal transmission. A synergistic effect of Ag^+^ ions and ROS is also considered: ions inhibit respiratory enzymes, facilitating ROS generation. It should be stressed that the concentration of ROS produced by AgNPs is too low for the oxidation of organic substances in water. When the contents of AgNPs and H_2_O_2_ are 2 and 10 ppb, respectively, the amount of ROS is 5–35 ppt at pH 7–10 [111]. However, this is enough for bacterial degradation.

### 4.2. MXenes and Advanced Carbon Nanomaterials

In contrast to AgNPs, the antibacterial effect of MXenes [113] and advanced carbon nanomaterials (CDs [114], carbon nanofibers [115], CNTs [116], graphene [44], nano-onions [117] and fullerenes [118]) is only due to ROS [44]. The activity of these nanomaterials is especially pronounced when bacteria adhere to them. 2D and 1D nanoparticles damage the cell membrane with their atomically sharp edges or needle-like ends (Figure 9b [44]). Punctures cause a leakage of the cellular contents and provoke oxidative stress. The action of nanomaterials strongly depends on their morphology and surface functional groups. For instance, single-walled CNTs possess higher antibacterial activity than multi-walled CNTs. The instability of the cell membrane caused by fullerenes is explained by their binding to peroxidase enzymes, which protect the cells from oxidative stress. The adhesion of nanoparticles is possible when their surface is uncharged or positively charged. For instance, GO flakes are negatively charged in weakly acidic, neutral and alkaline media; negatively charged bacteria are repelled by them. Thus, the antibacterial activity of filtration membranes containing advanced carbon nanomaterials and MXenes strongly depends on the solution pH. The same applies to membranes containing AgNPs, since the release of metal ions and bacteria adherence to nanoparticles are determined by the solution acidity.

### 4.3. Photocatalysts

Photocatalysis is an effective strategy, which allows one to reduce both biofouling and fouling with organic substances. Photocatalysts are inorganic semiconductors, a number of which are used in the processes of membrane separation, for instance, crystalline TiO_2_ (anatase), ZnO, CuO, WO_3_, BiVO_4,_ etc. [119]. Their electron–hole pairs are organized when these materials absorb the photon energy, which is equal to or higher than the energy of the band gap between the valence and conduction bands. An electron in the valence band crosses the band gap and appears in the conduction band:

Semiconductor + hv → e_cb_^−^(Semiconductor) + h_vb_^+^(Semiconductor). (18)

Here e_cb_^−^ and h_vb_^+^ are the conduction band electron and valence band hole, respectively. Further ROS, specifically •OH radicals, are formed at the semiconductor surface:

Semiconductor/H_2_O+ h_vb_^+^ → Semiconductor/•OH + H^+^. (19)

Additionally, •O_2_^−^ species and atomic oxygen are generated according to reactions (7) and (10) respectively; they were detected in [69]. An indirect method, which involves a photocatalyst applied to a substrate, allows one to estimate the ROS generation rate via the quantitative analysis of H_2_O_2_ (the result of reaction (8)) [120]. The rate achieves up to 40 µmol cm^−2^h^−1^; this is enough not only for bacterial degradation but also for the destruction of organic substances. Oxidation is accompanied by the formation of intermediate compounds. Ideally, oxidation occurs down to CO_2_, but this does not always happen. In contrast to AgNPs, photocatalytic bacterial degradation is caused only by ROS [121]. However, it should be stressed that both photocatalysts and AgNPs produce ROS themselves. In the case of MXenes and advanced carbon nanomaterials, ROS are generated only due to cell damage. Additionally, AgNPs poison cells with Ag^+^ ions, which are released due to metal corrosion.

## 5. Membranes Containing Non-Destructive Inorganic Modifiers

### 5.1. Natural and Synthetic Aluminosilicates

Significant advantages of natural aluminosilicates over other modifiers are their availability and low cost. Zeolites can also be obtained via “green” hydrothermal synthesis. Membranes containing aluminosilicates are manufactured by phase inversion [76,91,92,99,122,123] or electrospinning [93,124].

Clay minerals such as halloysite (preliminarily functionalized with dextran) [92] and palygorskite have been used as modifiers [91,92]. Their primary particles are nanorods whose diameter is up to ≈50 nm (Figure 10a,b). Compared with polymer membranes, the active layer of the modified materials contains larger pores (Figure 10c,d [92]); these provide a threefold increase in PWP [91] (Table 2). This increase can also be caused by the hydrophilizing effect of the clay particles. The retention of PVA (70 kDa) [91] or BSA [92] slightly reduces. No effect of the particle length on the permeate flux and BSA retention has been found [92].

In contrast to [91,92], nanocrystallites of clinoptilolite functionalized with a surfactant reduce the porosity (from 80 to 69%) and pore size (from 6 to 4 nm) of PVDF membranes [122]. This results in a 2.5-fold decrease in PWP despite the higher hydrophilicity of the modified material, which effectively retains an anionic dye. Membranes containing clinoptilolite were tested for the treatment of synthetic graywater containing low- and high-molecular-weight substances. Chemical oxygen demand (COD) and turbidity decreased by 63% and 97% respectively. Natural zeolites deteriorate the performance of the PVDF membrane, but the composite of this zeolite with functionalized silica slightly increases both PWP and Methylene Blue retention [99]. Bentonite combined with functionalized silica increases the PWP sixfold and improves the retention of humic acid by the PAM membrane [100]. However, the pristine material also shows high retention (90%).

Both natural [93] and synthetic zeolites [76] sufficiently improve the retention ability of polystyrene [93] and PES [76] membranes for NaCl [93] and Pb(NO_3_)_2_ salts [76]. The removal of Pb(NO_3_)_2_ from water was performed at only 1.6 bar, as opposed to commercial nanofiltration membranes. The highest retention (97%) is achieved at pH 6. At an elevated temperature, the retention is lower, evidently due to enlarged pores. The addition of synthetic zeolite to the polymer reduces the MWCO for the PVDF membrane from 69 (BSA) to 35 kDA (pepsin) [123]. The membrane made of this polymer, which contains natural zeolite, removes turbidity from graywater almost completely, but the COD removal is sufficiently lower [124].

In general, the addition of aluminosilicates to filtration membranes ensures an insufficient increase in retention except [76,124], and even causes its decline [91,92]. Nevertheless, these membranes could be useful for concentrating macromolecular compounds, taking into consideration their antifouling resistance (the *FRR* parameter reaches up to 96% [122]).

### 5.2. Phosphate and Oxides of Multivalent Elements

Phosphates and hydrated oxides of multivalent elements can be easily obtained from available, relatively non-hazardous and inexpensive reagents; their surface can be readily functionalized. These materials are widely used to manufacture polymer–inorganic membranes for low-temperature fuel cells to provide charge transport at an elevated temperature when proton-conducting polymers are dehydrated [125]. Membranes containing these modifiers are also applied to electrodialysis [126]. Zirconium phosphate is a cation-exchanger due to its hydro- and dihydrophosphate groups [127]. Hydrated oxides possess amphoteric properties: surface hydroxide groups are dissociated in alkaline solutions and protonated in acidic media. Thus, they are cation or anion exchangers depending on the solution pH [128,129,130].

Zirconium compounds such as phosphates [101,131,132,133] and hydrated oxides [19,67,68,70,133] have been used for the improvement of the separation ability and antifouling resistance of a number of polymers. Amorphous zirconium phosphate was embedded into the pores of commercial membranes by their impregnation with a sol of Zr hydroxocomplexes followed by deposition with H_3_PO_4_ [131,132,133]. The nanoparticles obtained in this manner were also fixed on the membrane surface with gelatin [101]. Hydrated zirconium dioxide was deposited in ammonia vapor [19,67,68,70,133]. The problem was to localize the modifier mainly in the active layer. A theoretical approach, specifically the Ostwald–Freundlich equation, was applied [133]:

(20) $$\ln\frac{C}{C_{\infty }}=-\frac{\beta v_{m}\sigma \cos\varphi }{RTr},$$

where *C* is the concentration of a compound, *C*_∞_ is its content in a saturated solution, *β* is the shape factor of particles, *v*_m_ is the molar volume of a compound, *σ* is the surface tension of a liquid, *φ* is the solvent contact angle, *r* is the particle radius, *R* is the gas constant, and *T* is the temperature. Thus, smaller particles are formed at a reduced temperature; they can deposit inside both macroporous substrate and the active layer. For crystalline materials, the molar volume is 21.7 (ZrO_2_) and 91 (α-modification of zirconium hydrophosphate, Zr(HPO_4_)_2_•H_2_O) cm^3^ mol^−1^. The molar volume of amorphous ion exchangers is assumed to be close to the values of crystalline materials.

Thus, hydrated zirconium dioxide is deposited both inside the active layer and the macroporous support of MF and UF membranes [19,67,133], whereas zirconium hydrophosphate is accumulated mainly in the support [133]. During precipitation of the modifiers inside the pores of symmetric MF membrane, primary nanoparticles form aggregates, which block macropores [131,132]. Pores between these nanoparticles determine the separation ability of polymer–inorganic membranes. Inorganic particles protect membranes against fouling; as a result, only their surface is contaminated. Conversely, pristine membranes show irreversible fouling. The same effect has been found for the membranes with an active layer during the filtration of pesticides [19,68].

Crystalline metal oxides [64,134,135], unfunctionalized [86,87,136] and functionalized silica [85,86,87,136,137] are blended with the polymer solution, and then membranes are formed via phase inversion. Another approach is to add an inorganic constituent (γ-alimina, gibbsite or boehmite) to water, where the PVDF membrane is formed [122]. Oxide nanoparticles are centers for the growth of hydrated aluminum oxide from the solution of Al_2_(SO_4_)_3_ salt. The resulting nanoparticles (30–60 nm) are located mainly on the outer membrane surface (Figure 11). Both amorphous and crystalline oxides of multivalent metals significantly affect the hydrophilic properties of polymers. For instance, TiO_2_ of anatase structure is considered mainly as a photocatalytic additive; however, its hydrophilizing effect is noted [64], and rutile also enhances hydrophilicity [134], though it is obtained at a higher temperature.

Silica is also a common modifier for filtration membranes due to its excellent hydrophilizing ability. For instance, a threefold increase in the permeate flux compared with the pristine PES membrane, and very high *FRR* parameter (97%) are reported [136] (Table 3). Silica functionalized with polyethyleneimine (PEI) provides complete retention of BSA and dyes [137]. Moreover, modified PES membranes show NF [86] and even RO [87] properties, since hardness ions are removed from water almost completely [86], and a high retention degree is achieved even for NaCl [87].

Oxides and phosphates of multivalent elements enhance the antifouling resistance of polymer membranes and significantly improve the retention of both low- and high-molecular-weight compounds. Some polymer membranes cannot retain inorganic salts [86] or pesticides [19]; however, retention can exceed 90% after modification. The problem is the degradation of metal oxides in acidic solutions used for rinsing the membrane stacks. However, anatase-containing membranes are capable of self-cleaning due to their photocatalytic properties.

As a rule, the attention is not focused on modifier leakage from the polymers. Regarding the particles embedded in pores, this leakage can be reduced by their adhesion to the pore walls due to the roughness of the aggregates of nanoparticles [133]. Since acidic solutions are used for modification, partially hydrolyzed PAM and PAN can perform the function of a “glue” [19]. Nevertheless, long-time experiments were not conducted. It should be noted that model solutions were used in all works mentioned in this subsection except [70,133], where corn distillery [133] and milky whey [70] were filtered.

### 5.3. Magnetic Nanoparticles

Magnetic nanoparticles grafted to the membrane surface stir the hydrodynamically immobile layer of the solution at the membrane surface under the influence of a magnetic field. Thus, concentration polarization decreases; as a result, membrane fouling is depressed [47,138,139].

The modification of the surface of PS [47] or PVDF [138,139] membranes with magnetic Fe_3_O_4_ nanoparticles is performed as follows. Linear PEI spacers are grafted onto the activated surface. Magnetic nanoparticles (<50 nm) functionalized with polyacrylic acid (PAA) are attached to the spacers (Figure 12, [47]). They are fixed on the surface due to the interaction between oppositely charged amino and carboxyl groups. When the BSA solution is filtered through the modified membranes, Darcy law is obeyed, indicating their antifouling resistance. Besides the depression of concentration polarization, magnetic nanoparticles together with spacers hydrophilize the membrane surface [138] (see Table 3). The MWCO is 8 kDa for polyethylene glycol [139]. The membranes were used for protein fractionation [138] and for the removal of Fe^2+^ ions from water [139]. Preliminarily, inorganic ions were bonded with PAA; their complete retention is achieved.

In contrast to [47,138,139], magnetic nanoparticles (BaFe_12_O_19_ [140] and Fe_3_O_4_ [141]) were incorporated into the pores of PS, PAN [140] and PES UF membranes [141]. The modification method involves the deposition of a pre-synthesized magnetic component together with hydrated zirconium dioxide, which is formed directly in the membrane pores [140]. Another approach involved mixing the polymer with magnetic nanoparticles functionalized with carboxymethyl chitosan, followed by the membrane formation [141]. The membranes exhibit high retention of vegetable proteins from sugar beet juice (up to 87%) due to the sieving effect [140]. Lower retention (up to 77%) was found for the membranes containing only hydrated zirconium dioxide. The membrane modified with functionalized magnetite was used for filtering activated sludge; the *FRR* parameter was 89% [141]. This material is recommended for MBRs.

The problem with membranes containing magnetic nanoparticles is their degradation in acidic solutions. This complicates the cleaning of membrane stacks. However, the stability of the surface particles at 2 bar has been reported—only 5% of the inorganic modifier is removed over 10 h [139]. The movement of grafted particles in a magnetic field can be complicated by the swelling of hydrophilic spacers.

## 6. Membranes Including Destructive Modifiers

### 6.1. Advanced Cabon Nanomaterials

#### 6.1.1. Common Methods for Synthesis of Carbon Modifiers

Several allotropic carbon modifications (CDs, CNTs, nanofibers, GO, rGO, fullerenes and nano-onions) are applied to the modification of MF and UF membranes. Advanced carbon nanomaterials as well as graphitic carbon nitride (g-C_3_N_4_) are also used to prepare photocatalysts for membrane applications.

GO (in fact, a graphene-like nanomaterial) is obtained by the exfoliation of graphite layers using a chemical method developed by Hammers [142]. A very promising approach is the opening of CNTs with chemical, physical and electrochemical techniques [143]. The removal of oxygen-containing functional groups from GO allows one to obtain rGO [144]. Key design factors involve the C/O ratio in the final product, the removal of one type of oxygen group (only hydroxyl, or only carbonyl, or only epoxy), the healing of the GO surface defects from oxidation, and the choice of “green” reducing agents. Thermal reduction is performed in an oxygen-free medium or by heating the GO in a microwave oven. Other approaches are photocatalytic or electrochemical reduction.

Arc discharge, laser ablation and chemical vapor deposition are used to synthesize CNTs [145]. The first two methods involve solid precursors as a source of carbon; they are evaporated at thousands of degrees Celsius. These techniques allow one to obtain high-quality nanotube structures despite the large amounts of by-products. Chemical vapor deposition uses hydrocarbon gases and metal catalyst particles as a “seed” for nanotube growth at 500–1000° C. Arc discharge and laser ablation can produce single-walled CNTs with a mean diameter of ≈1.4 nm. The synthesis of such 1D nanomaterials, such as carbon nanofibers, involves obtaining biocellulose followed by its drying with liquid nitrogen to form an aerogel, and its pyrolysis in an inert atmosphere [146].

Unlike nanofibers, CDs can be formed at much lower temperatures, but elevated pressure is required (hydrothermal synthesis) [146,147,148]. This method is considered a “bottom-up”; it involves the polymerization and carbonization of organic molecules. The synthesis of CDs requires cheap and available reagents, for instance, ethylenediamine and citric acid [148], or feedstock of biological origin (for instance, polysaccharides) similarly to nanofibers [146,147]. However, these carbon 0D nanomaterials obtained with the hydrothermal technique must be purified from by-products. CDs are also synthesized with the “top-down” techniques: arc discharge, laser ablation, and chemical or electrochemical oxidation from graphite, graphene, nanotubes and polymers [147].

The most common methods for the synthesis of C_60_ fullerene are the resistive arc heating of graphite and the laser irradiation of carbon or polyaromatic hydrocarbons [149]. These approaches allow one to obtain large amounts of fullerenes. Small nano-onions (multilayered fullerenes, 5–8 carbon shells) are produced by converting nanodiamonds using heating, electron irradiation, arc discharge between graphite electrodes in water or liquid nitrogen, or by naphthalene pyrolysis [150]. Large nano-onions (up to 30 nm) are obtained by the pyrolysis of wood wool followed by treatment with concentrated nitric acid. To manufacture g-C_3_N_4_, the polycondensation of cyanamide, dicyandiamide, melamine and all other C/N substances can be applied [151]. For instance, cyanamide is transformed into g-C_3_N_4_ at 550° C in an atmosphere of air or inert gas.

It should be stressed that the methods of synthesis of advanced carbon nanomaterials are ecologically dangerous and require high energy consumption. However, CDs can be synthesized with a “green” method; similarly rGO can be obtained from GO. Below, we briefly consider membranes modified with advanced carbon nanomaterials. More detailed information was systematized earlier [34,152].

#### 6.1.2. GO

A common method for modifying MF or UF membranes with GO is layer-by-layer self-assembly onto the outer surface. GO flakes are connected edge-to-edge and layer-by-layer, forming sheets (Figure 13a [153]). Two-dimensional nanochannels between them are responsible for fluid transport. The small size of the channels and the high surface charge of the flakes provide the retention of low-molecular-weight compounds and ions. However, the layers are destroyed by flow due to the hydration of functional groups, their dissociation and electrostatic repulsion. Defects on the periphery of the flakes cause pores that cannot ensure the retention of small species.

To solve these problems, GO is combined with hydrophilic polymers: PEI [94], chitosan [154], and PVA [95]. The self-assembly method involves chemical activation of the membrane surface and its modification with a polymer, followed by the attachment of the GO flakes. However, the loops of the polymer chains disorder the flakes, widening pores and making them more tortuous [155]. Tannic acid is polymerized on the surface of the membrane and GO flakes, forming short chains (Figure 13b) [153]. MF membranes were modified via:

- The deposition of a GO/tannic acid composite from mixed solutions [63];
- The formation of the GO/tannic acid/PEI composite (the amino groups of PEI interact with the catechol groups of tannic acid in alkaline media), where the GO flakes are inside the cross-linked polymer [156];
- The organization of an intermediate PEI layer between the membrane surface and the layer of the GO/tannic acid composite [157]. To minimize GO leakage, the order of the layers is tannic acid/GO/tannic acid [158].

The PS membranes coated with the GO/tannic acid composites show significantly higher WCAs compared with the pristine membrane (62°), in contrast to composites with other polymers (Table 4). This can be explained by the partial GO reduction with tannic acid, which is transformed into a quinone form [159]. Moreover, tannic acid was deposited under aggregation conditions (pH 3). Thus, phenolic groups are exposed mainly inside aggregates providing hydrophobicity to the membrane surface. The pores of the layer are evidently hydrophilic since the *FRR* parameters are rather high. After modification in a neutral buffer, when functional groups of tannic acid are dissociated, PES membranes demonstrate enhanced hydrophilicity (WCA ≈ 15°) [156] probably due to the absence of its aggregation. The membranes were applied to the removal of hexane (96% retention) [156] or dyes [63,94,153,154,158] from water, and also to the recovery of α-tocopherol from the extract of soybean oil residues (in this case, low permeability was achieved, 0.5 L m^−2^h^−1^bar^−1^) [157] or an enzyme from the extract of flax seeds [160]. Colloidal particles are retained due to sieving, and electric exclusion can contribute to dye concentrating since salt additions depress the retention of their ionic forms. GO was also embedded into the pores of PAN membranes together with hydrated zirconium dioxide [80]. Irreversible fouling was minimized in this manner; the removal degrees of OVA and BSA were 95 and 98%, respectively.

Filtration materials were also obtained by blending GO with polymers, followed by membrane formation [79,89,90,98,161,162,163]. An experimental design was applied to the preparation of MF membranes; the material obtained under optimal conditions showed the highest antifouling resistance [161]. 

The membranes in which GO is distributed in the polymer were used even for the removal of Pb^2+^ ions from water [45,98] and blood plasma [45]. They show antimicrobial activity despite the absence of AgNPs or photocatalysts [91,96]. However, no cytotoxic effect was observed for the membrane containing a laponite–GO composite [98]. Instead, it enhances the proliferation of human epidermal keratinocyte cells.

As seen from Table 4, the membranes obtained by layer-by-layer self-assembly are characterized by higher retention for dyes than the materials prepared by the blending technique. However, only planar membranes were manufactured via surface modification and tested using dead-end modules (cross-flow filtration was reported probably only in [164]). This modification method was not applied to the materials for tubular modules, which are more efficient than flat-and-frame devices.

#### 6.1.3. rGO

The disadvantage of GO is particle aggregation due to hydrogen bonds between functional groups. These result in the formation of impermeable areas, which negatively affect membrane performance. Instead of GO, rGO is sometimes used for modification. The reduction was carried out using a hydrothermal method [165,166], ascorbic acid [167], ultraviolet irradiation [168], or with inorganic reducing agents (N_2_H_4_⋅H_2_O or NaBH_4_) [169]. The interplanar distance in GO decreased from 0.92 to 0.38 nm during hydrothermal treatment, but long-time reduction caused cracking of the flakes [165].

Polymer macroporous membranes were coated with rGO without cross-linking agents [165,167,168,169]. However, a MOF was used for cross-linkage; increasing its content improved the membrane performance [166]. The rGO layer with the maximum cross-linkage retains not only dyes, but also the salts of heavy metals (Table 5). During the filtration of a Methyl Blue solution through the membrane containing cracked rGO, the permeate flux is 20 times higher compared with the pristine material, but the retention decreases [165]. Uncracked rGO shows better performance. The maximum values of PWP and Aniline Blue retention are achieved at a certain thickness of the rGO layer [168].

As seen from Table 4 and Table 5, the membranes coated with rGO are characterized by higher PWP than those modified with non-crosslinked and even crosslinked GO, despite their stronger hydrophobicity. This is due to the foam-like structure of the rGO layer; at the same time, GO forms a more compact film. However, the retention of the ionic forms of low- molecular-weight compounds is lower due to weaker electrical exclusion caused by the lower content of O-containing groups [167,168]. Nevertheless, higher dye retention is reported specifically for the rGO membrane [167]. As a rule, the information about antifouling resistance is lacking for materials of this type.

#### 6.1.4. 1D Nanomaterials

Several filtration membranes were obtained by modifying polymers with 1D nanomaterials, such as CNTs or carbon nanofibers. Hollow-fiber PVDF scaffolds were prepared by electrospinning; the fibers were coated with an active layer consisting of a carbon constituent and PAN as a binder [81]. An active layer (chitosan/CNTs) was formed on the surface of a PS membrane [170]. Membranes were also obtained by blending CNTs with PES [171,172] and chitosan [173]. Surface modification via the layer-by-layer deposition of CNTs should also be mentioned [174]. The PS membrane was activated with plasma; further the CNTs layers were alternately deposited on the surface, and up to 20 bilayers were formed. In contrast to [81,170], no binder was used. Carbon nanofibers (10 nm thick) were also applied to the surface of a UF PVDF membrane without a binder (Figure 14) [175].

CNTs were preliminarily functionalized by oxidation with a mixture of HNO_3_ and H_2_SO_4_, -OH and -COOH groups were formed at the ends of the nanotubes [81,170,171]. Both oxidized and aminated CNTs were employed [174]. To provide ordered CNTs and concentrate them on the surface, they were functionalized with Ni nanoparticles, and the PES/CNTs casting solution was exposed to a magnetic field [172]. The chitosan-based membrane was additionally modified with iodine to enhance its antifouling resistance [173]. Iodine disrupts the intermolecular hydrogen bonding networks of chitosan, but no its interaction with the alkyl fragments of the polymer chains was detected.

Compared with pristine polymers, CNTs increase both PWP and permeate flux ≈3–4-fold [81,170]; a PWP increase of even sevenfold is reported for the membrane in which the CNTs are concentrated specifically at the surface [172] (Table 6). In the latter case, colloidal particles are retained almost completely, in contrast to the pristine material and the membrane in which CNTs are uniformly distributed in the polymer. Very high permeability is reported in [81] for the membrane containing oxidized CNTs, but only 30% of COD is removed from surface water (nevertheless, the retention of turbidity reaches 93%). Membranes modified with oxidized CNTs show better performance than the polymer containing non-oxidized nanotubes [170]. Conversely, oxidized and aminated CNTs, which are deposited one after another, depress permeability but significantly improve the retention of low-molecular-weight compounds [174]. This is caused by the coupling of CNTs with oppositely charged functional groups. Carbon nanofibers on the membrane surface also enhance retention [175]. However, the solution permeability increases, in contrast to [174]. Oxidized CNTs provide high removal of heavy metal ions from an industrial solution; the highest retention was found for FeCl_3_ probably due to the precipitation of insoluble hydroxy compounds. Chitosan-based composites containing oxidized or non-oxidized CNTs transform the polymer membrane into an NF material [170].

Modifying CNTs with iodine opens additional perspectives, although a deterioration in the retention ability of the resulting membranes was reported [173]. Besides high permeability, membranes containing CNTs/iodine can inactivate bacteria almost completely. The highest activity against Gram-positive bacteria has been found. Nevertheless, these membranes show lower dye retention than the polymer modified with pure CNTs.

#### 6.1.5. Fullerenes and Nano-Onions

In contrast to GO, rGO and CNTs, hydrophobic non-functionalized fullerenes are not common modifiers for filtration membranes. Nevertheless, composites of the C_60_ fullerene with polyethylene oxide were considered [77]. Modification increases the pore size compared with the pristine polymer, though fullerene acts as a cross-linking agent. This negatively affects the retention of BSA, OVA, γ-globulin, and other macromolecular compounds. Fullerene-containing membranes based on poly (2,6-dimethyl-1,4-phenylene oxide) were applied to the purification of water from hormones [176]. Their removal is due to adsorption on the membrane. The highest retention (99%) was found for the material containing 2% of fullerene; a higher amount decreases estrogen retention due to pore widening.

Polymers modified with functionalized fullerenes and nano-onions show better performance. PVDF membranes containing fullerene functionalized with a polyethylene glycol/polypropylene glycol copolymer exhibit the retention of bromelain, OVA and BSA (up to 99%) [177]. Nano-onions functionalized with sulfonyl groups (Figure 15) were used for modifying PES membranes [78]. They enhance hydrophilicity—the WCA decreases from 88° (pristine membrane) down to 31° (modified membrane). After the filtration of the BSA solution through the pristine and modified membranes, the *FRR* parameter reaches 57% and 94% respectively. Modified membranes show a retention of 98%.

#### 6.1.6. CDs

The advantages of CDs over other carbon nanomaterials are as follows [147]:

- The possibility of obtaining them via a “green” hydrothermal method from cheap and available feedstock;
- Functionalization with hydrophilic groups directly during synthesis; these groups improve compatibility between CDs and the polymer via electrostatic interactions and hydrogen bonding;
- Good dispersibility in casting solutions due to their ultrasmall size. This means the uniform distribution of CDs in the bulk of the polymer. CDs can also be embedded into the pores of polymer membranes together with another modifier [70].

Since CDs are very small, they are applied mainly to the modification of NF and RO membranes. Nevertheless, some CD-containing MF and UF membranes have been obtained (Table 7). The nanoparticles were ordered in the casting solution using a direct electric field [74]. Compared with the pristine polymer, the membrane shows better performance, particularly the PWP value is 2.6 times higher, and the irreversible fouling is only 5%. However, the retention ability towards BSA is slightly deteriorated due to an increase in the pore size from 25 to 33 nm. Narrower pores are evidently formed in the polymer film, which contains CDs functionalized with β-cyclodextrin (βCDx), than those in the composite with unfunctionalized CDs (the PWP is lower by an order of magnitude) [178]. Probably βCDx acts as an additional cross-linking agent during membrane formation. A similar effect is attributed to the membranes whose pores are filled with a CD-containing composite [70]. Nevertheless, the membranes modified with CDs-βCDx demonstrate excellent antifouling stability (reversible fouling dominates) [178]. It is interesting that during the repeated filtration of the BSA and Na alginate solutions, an increase in antifouling resistance was found. In the case of humic acids, the resistance becomes weaker.

The effect of the surface groups of the modifier on membrane performance is ambiguous: CDs with nitrogen-containing groups (mainly amino groups) depress permeability but slightly improve antifouling stability [179]. Opposite results were obtained in [180], where both amino and sulfo groups enhance modifier efficiency. The combination of such antibacterial modifiers as AgNPs and CDs, which simultaneously contain -COOH, -OH, -NO_2_, -NH_2_ and -SH groups, makes it possible to obtain membranes with strong activity against bacteria [181]. The materials are especially effective against Gram-positive bacteria. The modifier reduces pore branching, facilitating fluid transport (Figure 16). Membranes were applied to oil/water separation: the organic liquid is almost completely removed from water, and the *FDR* parameter is 50%. A membrane modified with CDs/AgNPs composite removes only 50% of color, but it shows good antifouling performance [182].

Due to their hydrophilizing effect, CDs significantly improve the resistance of polymer membranes to fouling, and, in many cases, enhance water permeability. When CDs are blended with polymers prior to membrane formation, their effect on retention is insufficient; sometimes even a decrease is reported [74]. Probably the cross-linking ability of CDs is limited by their size. Nevertheless, the functionalization of CDs or their combination with other inorganic modifiers opens additional perspectives.

### 6.2. MXenes

2D MXene materials originate from 3D MAXenes, the formula of which is M_n+1_AX_n_ (M is Sc, Ti, V, Cr, Mn, Zr, Nb, Mo, Lu, Hf, Ta) [183]. A list of the A elements includes Al, Si, P, S, Ge, Ga, As, In, Sn, Tl, and Pb; the X elements are C or N. 3D compounds are delaminated by etching the A phase (carbide or nitride) with HF (Figure 17a [184]). To prevent a violent reaction, HCl is added to LiF for the gradual HF release. Etching results in functional groups (fluoride, carbonyl, hydroxyl) on the surface of MXenes (denoted as T); thus, the M_n+1_X_n_T_x_ composition is attributed to MXenes. Hydroxyl groups provide their hydrophilicity. This property, as well as the 2D structure, makes it possible to use MXenes for membrane modification.

Methods for obtaining MXene-containing membranes involve layer-by-layer deposition, electrospinning, and phase inversion. When MXenes are blended with polymers prior to membrane formation, an ambiguous effect of the modifier on membrane performance is noted (the optimal amount is from 0.1% to 10% [185]). Similarly to GO and rGO, the MXene flakes form nanochannels on the outer membrane surface. Water is transported through these nanochannels, but other molecules are retained. Microporosity demonstrates growth: for instance, these values are 0.28 cm^3^g^−1^ (pristine polymer) and 0.78 cm^3^g^−1^ (modified membrane), and the mean pore size decreases from 13 to 2 nm. However, the measurements were carried out for the dry material; the porous structure of MXenes changes in aqueous media due to swelling. This is especially important when the inorganic constituent is distributed in the polymer. This disadvantage is minimized by a small content of the modifier; for instance, its optimal amount is only 0.05% [73].

To enhance resistance against organic substances and biofouling, MXenes are combined with hydrophilic (GO) [186] or antibacterial (AgNPs) [187] components. The distance between the layers of Ti_3_C_2_T_x_ and GO is 14.28 Å [188]. When the composite swells in water, the distance increases by 5 Å (2 layers of water molecules). As opposed to the loose polymer membrane and friable MXene coating, MXene forms a dense layer together with GO (Figure 17b–d). This layer provides the high retention of dyes (Table 8). However, the retention growth is inconsiderable compared with the membrane coated with pure GO. This is probably due to poor coupling between the flakes containing similarly charged groups and also due to the different swelling of MXene and GO. Membranes coated with an MXene/AgNPs composite retain not only dyes but also inorganic salts (however, the membrane resistance against biofouling is outside the scope of the attention) [187]. Due to strong cross-linking ability, MXene flakes distributed in the polymer are responsible for the formation of tortuous pores [182]. This provides oil/water separation, since pores block the oil droplets due to sieving. The membrane hydrophilicity reduces oil adhesion.

Antifouling resistance of the membranes against low-molecular-weight compounds, for instance, Methylene Blue, depends on the solution composition [186]. A low *FDR* parameter (10%) has been found at pH 1 (no dye protonation and surface charge of MXene, and, as a result, very low adsorption). This value increases up to 66% at pH 8 due to the adsorption of protonated dye molecules on the negatively charged MXene flakes.

As follows from Table 8, MXenes increase the PWP value by ≈2–3-fold compared with pristine membranes. Very high PWP (>1000 L m^−2^h^−1^bar^−1^) [75,189] has been found for membranes whose polymer constituent is also characterized by high fluid permeability. As opposed to the pristine membrane, the modified material is able to retain not only humic acid [190] but also dyes [191]. In all cases, MXenes also enhance antifouling resistance, since the *FRR* parameter exceeds 50%. However, it is less than 100% indicating irreversible fouling and the necessity to use chemical reagents for membrane cleaning (the deterioration of membrane performance without chemical regeneration is reported, for instance, in [186]). Probably this is a reason for the usage of only model solutions to test the membranes. Though titanium carbide is chemically inert, chemical instability of MXenes in aqueous media, especially in acidic solutions, is pointed out [192]. Additionally, the high cost of MXenes (several hundred USD per 1 g) is a barrier against commercialization of membranes modified with these 2D nanomaterials.

**Figure 17:**
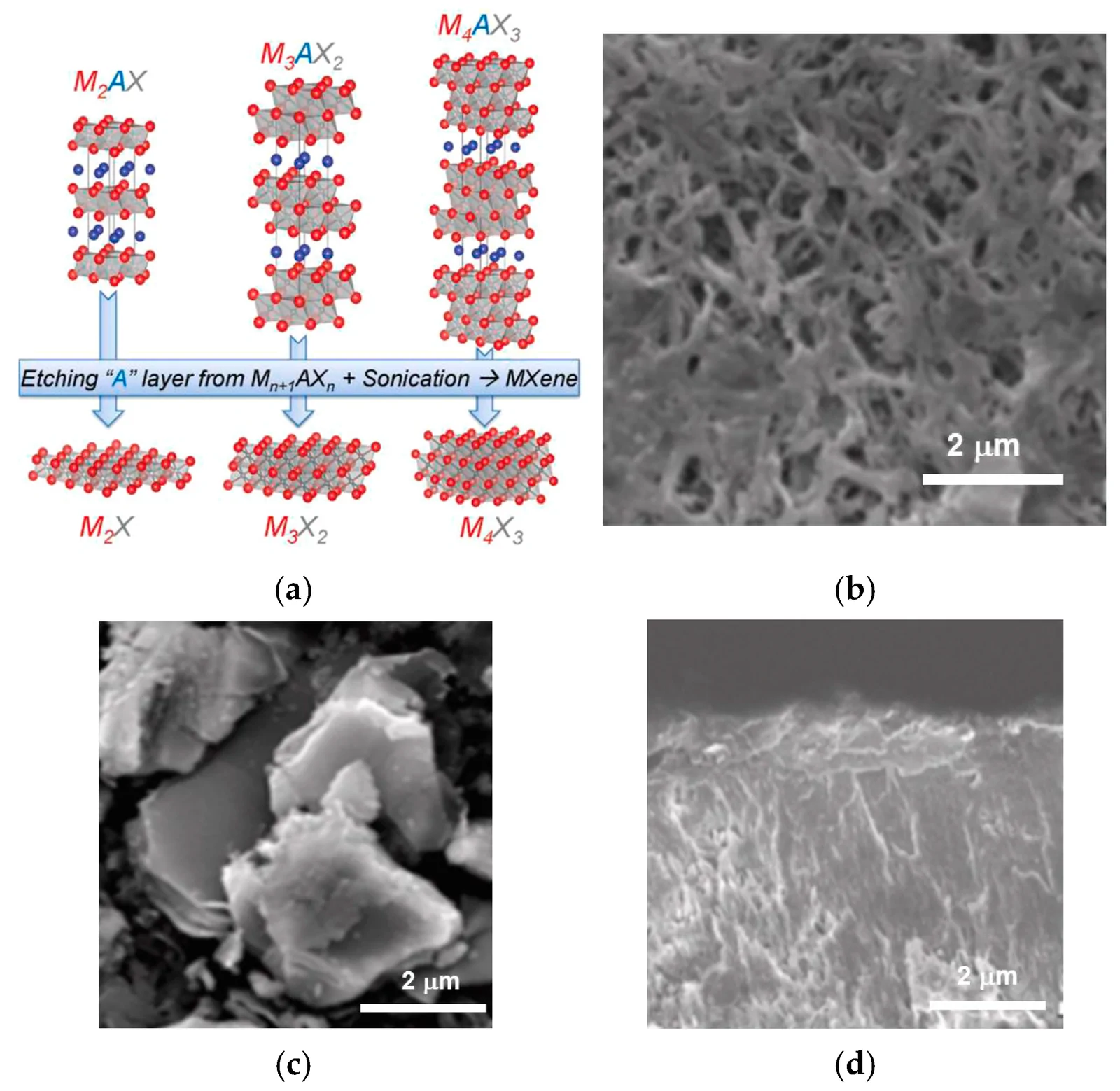
(**a**) Delamination of MAXenes [184]; (**b**) SEM image of outer surface of pristine polyphenylene sulfide MF membrane; (**c**) MXene; and (**d**) MXene/GO coating [186]. Reproduced with the permission from Elsevier.

**Table 8:** Functional properties and performance of the membranes modified with MXenes.

Polymer	Modifier	WCA (Degree)	Filtration				Ref.
			PWP (L m^−2^h^−1^bar^−1^)	Solution			
				Component	Retention (%)	*FRR* (%)	
Acetylcellulose	-	-	350	dye, BSA	50, 83	48	[185]
	Ti_3_C_2_T_x_ ^1^	-	522		97, 99	65	
Polysulfate	-	94	270	BSA	-	81	[73]
	Ti_3_C_2_T_x_ ^1^	88	685		97	90	
PES	-	75	1000	oil/water	95	40	[75]
	Ti_3_C_2_T_x_ ^1^	65	2300		98	65	
Polyphenylsulfone	-	83	45	dyes, humic acids	-	67	[190]
	V_2_CT_x_ ^1^	71	123		82, 98	75	
PVDF	-	75	350	-	-	-	[191]
	Ti_3_C_2_T_x_ ^1^	38	550	dye	99	86	
PVDF		70	400	oil/water, PAC	23, 25	75	[189]
	Ti_3_C_2_T_x_ ^1^	65	1250		50, 85	92	
Polyphenylene sulfide	GO/MXene ^2^	46	216	dye	>99	>85	[186]
PES	AgNP_s_/Ti_3_C_2_T_x_ ^2^	39	30	dyes, salts ^3^	≈100, 57–84		[187]
Acetylcellulose	GO ^2^	37	-	dyes	65–100	-	[188]
	Ti_3_C_2_T_x_ ^2^	62	-		40–95	-	
	GO/Ti_3_C_2_T_x_ ^2^	61	14		68–99	-	

^1^ Modifier is distributed in polymer; ^2^ Modifier is located on the membrane surface; ^3^ Salts: MgSO_4_, MgCl_2_, Na_2_SO_4_, NaCl.

### 6.3. AgNPs as Multifunctional Modifier

AgNPs are synthesized by the chemical reduction of Ag^+^ ions using synthetic and natural reagents, for instance, NaBH_4_ (the reactions occur under cooling) [193]:

2AgNO_3_ + 2NaBH_4_ → 2Ag + H_2_ + B_2_H_6_ + 2NaNO_3_; (21)

B_2_H_6_ + 6H_2_O →2H_3_BO_3_ + 6H_2_ (22)

Another widely used reagent is sodium citrate (Na_3_C_6_H_5_O_7_), which acts at >70 °C:

Na_3_C_6_H_5_O_7_ + AgNO_3_ + H_2_O → Ag + CH_2_O + CO_2_ + NaNO_3_. (23)

Heating is also required in the case of flavonoids or alcohols. The reduction with saccharides and aldehydes is performed at a lower temperature (<50 °C). As opposed to the mentioned reagents, N_2_H_4_, H_2_SO_3_ and H_3_PO_2_ reduce Ag^+^ ions under ambient conditions; the nanoparticles are formed very quickly. The excess of the reducing agent stabilizes their colloidal solution. Depending on the synthesis conditions, AgNPs of a certain size and shape are formed; they are characterized by stronger or weaker antibacterial activity. For instance, this effect against Gram-positive and Gram-negative bacteria decreases in the order: spheres > disks > triangles [194]. However, the opposite effect would be expected since the sharp edges of the particles easily damage the cell membrane. Small AgNPs (<10 nm) show higher activity than larger particles (10–100 nm) [195]. For smaller AgNPs, bacterial degradation requires a shorter time and a less concentrated solution.

Modifying membranes with AgNPs is performed in different ways. Sometimes blending pre-formed AgNPs with a polymer solution is called ex situ modification [196]. As-prepared AgNPs are encapsulated in polymerized tannic acid and attached to the membrane surface [197], whereas capsule-free nanoparticles are fixed on the surface via cysteamine [65] or embedded into pores [198]. The in situ method provides the silver reduction directly in the polymer solution. The polymer and AgNO_3_ are dissolved in an organic solvent (for instance, in N-methyl-2-pyrrolidone), which is also a reducing agent [196]. Ag^+^ ions can also be reduced with dopamine [199] or dimethylacetamide [106].

AgNPs are concentrated in the pores of the membrane, which was obtained using the ex situ method; they are absent on the outer surface [196]. In the case of in situ modification, cubic AgNPs are formed on the outer surface, and spherical nanoparticles are uniformly distributed in the bulk of the polymer [106,196]. This morphology affects the antibacterial activity of the membranes, which inactivate bacteria almost completely [196]. As opposed to the membranes obtained ex situ, the effect of AgNPs content on antibacterial properties is marginal. During in situ modification, the PVDF polymer is transformed from the non-polar α phase to the polar β phase; bacteria are repelled by the polar surface, the hydration layer of which is a protector from bacterial adhesion [106]. AgNPs make an additional contribution to hydrophilicity due to the shell of reducing agents and reduction products on their surface. Thus, the membrane obtained in situ shows better performance (Table 9).

Membranes containing AgNPs, which were obtained by reduction with flavonoids, depress the development of both Gram-positive and Gram-negative bacteria; their antibacterial activity is similar to that of free nanoparticles [198]. These reducing agents provide the longest period of antibacterial action of AgNPs and membranes containing them (about 1 year) [31]. Membranes containing AgNPs were recommended for MBRs [82,200] and for the treatment of surface water [106] or oil-containing wastewater [181].

Besides the ability to destroy bacteria, AgNPs enhance the hydrophilicity of polymers. The hydrophilizing effect is due to the products of silver corrosion and also due to adsorbed reducing agents and reaction products. Hydrophilicity is reinforced by the combination of AgNPs with GO [29], MXenes [187], silica [200], TiO_2_ [201], or CDs [181,182].

**Table 9:** Functional properties and performance of membranes modified with AgNPs.

Polymer	Modifier	WCA (Degree)	Filtration						Ref.
			PWP, (L m^−2^h^−1^bar^−1^)	Ag^+^ Leakage (µg cm^−2^day^−1^)	Solution				
					Component	Retention (%)	*FDR* (%)	*FRR* (%)	
PS	-	70	31	-	Activated sludge	-	66	45	[82]
	AgNPs	56	33	168 *		-	50	40	
PS	-	-	-		BSA	40	-	-	[199]
	AgNPs	-	-	0.012		40	-	-	
PVDF	-	69	260	-	BSA/ COD of river water	93/23	51	63	[106]
	ex situ AgNPs	58	370	0.003		92/33	41	87	
	in situ AgNPs	53	430	<0.003		96/36	40	96	
PAM	-	67	2.5	-	NaCl	96	25	-	[65]
	thiol groups	65	3.7	-		94	19	-	
	thiol groups/AgNPs	35	3.5	0.5		93	22	-	
PS, PAM	tannic acid-Fe^3+^	55	0.7	-	NaCl	98	-	-	[197]
	AgNPs/tannic acid-Fe^3+^	45	2	0.2		98	-	-	
PVDF	-	142	7500	-	BSA		33	71	[29]
	AgNPs	126	12,500	0.5			46	90	
	AgNPs/GO	122	14,500	0.7			53	93	
PVC	-	67	41		dye	92	56	48	[201]
	TiO_2_	62	45			93	47	52	
	AgNPs/TiO_2_	55	55	3		99	40	65	
PVDF	-	75	47	-	activated sludge, COD	80	-	-	[200]
	AgNPs/SiO_2_	62	57	0.5		90	-	-	

* Short-time leakage.

AgNPs are probably the only type of inorganic modifiers whose leakage from membranes is studied carefully, probably due to the toxicity of Ag^+^ ions. Moreover, silver corrosion decreases membrane lifetime. The rate of Ag^+^ release rapidly decreases over time and reaches a constant value after several hours (Figure 18) [199]. Normally the constant rate (determined for a long time) is several µg cm^−2^day^−1^ (see Table 9). The membrane containing AgNPs [197] or their nanocomposites [201] loses 2–4% of silver. Note that attention is not focused on the leaching of AgNPs. The stability of adsorbed species on the nanoparticles (reducing agents and products of their oxidation) is also not considered.

Due to their high antibacterial activity, the main function of AgNPs is to minimize biofouling of filtration membranes. Additionally, their hydrophilizing ability provides membrane resistance not only against biofouling but also against fouling with organic substances. Thus, the modified membranes show higher permeability (even ≈twofold [29]), an elevated *FRR* parameter (up to 96% [106]) and a lower *FDR* (down to 20% [65]) compared with pristine materials. The retention growth is not very significant, especially for real solutions [82,106,200]. The long-term effect of AgNPs on biofilm formation was not investigated; the studies are limited to antibacterial tests of as-prepared membranes.

## 7. Photocatalytic Membranes

### 7.1. Organization of Photocatalytic Processes

The separation processes combining MF or UF membranes and photocatalysis can be arranged as follows (Figure 19, [202]):

- Filtration through a photocatalytic membrane under light irradiation;
- Immersion of a photocatalytic membrane in a solution;
- Suspension of photocatalyst particles in a liquid, which fills the module supplied with a conventional filtration membrane;
- A chain of isolated elements: a container with a solution where the photocatalyst is suspended, and a module with a conventional filtration membrane.

The first scheme requires a special module, which provides light irradiation of the membrane containing a modifier both on the surface (for oxidation) and inside the polymer (for separation). A synergistic effect of the polymer (sieving, adsorption) and the photocatalyst is possible. Membranes are irradiated only during filtration, only during regeneration, or during both filtration and regeneration. When membranes are purified with light instead of chemical reagents, they are considered self-cleaning materials.

The membranes of the second scheme contain active particles only on the outer surface, transparent solutions are needed for their irradiation. The same requirement applies to the third and fourth schemes. Another limitation of suspensions is the necessity of separating finely dispersed particles from the solution.

### 7.2. Membranes Modified with Photocatalysts

Due to strong catalytic activity, relative chemical stability, a rather long lifetime of electron–hole pairs, moderate cost and low toxicity, TiO_2_ in the form of anatase is the most common material for modifying polymers. It is active not only against organic substances but also against both Gram-positive and Gram-negative bacteria, depressing their viability ≈50-fold even under visible light conditions and enhancing the antibiofouling resistance of polymer-based membranes [203]. TiO_2_ was mentioned only as a hydrophilizing agent; its photocatalytic activity was not considered [64,88,134,201]. However, TiO_2_ is active only under UF irradiation due to its wide band gap (3.2 eV). The band gap can be reduced via the generation of structural defects in the lattice by doping with ions of some metals, for instance, Ag and Au [202]. The membranes containing doped TiO_2_ were investigated in [69,204,205]. Other photocatalysts, such as ZnO, Ag_2_O [206], WO_3_ [207,208], BiVO_4_ [208] and their heterojunctions with TiO_2_ [208,209], are also used.

Photocatalyst nanoparticles can be distributed in the polymer or located on the outer membrane surface. They are stabilized with dopamine [62,210] or tannic acid [204]. An intermediate carbon layer (to adsorb the by-products of degradation) is deposited between the membrane surface and the photocatalyst [211]. Carbon can be added together with TiO_2_ to the polymer solution before membrane formation via electrospinning [203]. The nanoparticles can also be grafted to the polymer, for instance, via PAA [64].

The membrane performance is improved by combining photocatalysts with MXenes [207,212,213], GO [62,105,214] rGO [215], g-C_3_N_4_ [216] and CNTs [209] (Table 10). These heterojunction structures promote the separation of photogenerated electrons and holes and inhibit their recombination. Moreover, MXenes and carbon nanomaterials adsorb organic substances, enhancing both their retention and degradation. Photocatalytic activity is noted even for membranes containing only MXene [213,216].

Membranes are irradiated with visible or UV light during filtration, regeneration or simultaneously during filtration and regeneration as noted in Table 10. Additionally, regeneration combines rinsing and irradiation, or membranes are irradiated after rinsing. The removal of organic substances from solutions is due to both retention (in darkness) and degradation (under irradiation). A high removal degree (≥90%) is achieved in [69,208] specifically due to photocatalysis. Compared with the pristine membrane, this value grows significantly; however, it remains lower than 90% [62,206,210,211] especially in the case of turbid industrial wastewater [62] or river water [211]. One reason for this can be photocatalyst poisoning with organic substances, particularly with by-products of photodegradation. Indeed, the solution is decolorized, but the COD removal is not high, specifically due to by-products [210]. Also, the photocatalytic activity of the membranes becomes weaker from cycle to cycle of filtration–regeneration with water and light. For instance, the removal degree of tetracycline reduces by 7% after the 3rd cycle [216].

Modifiers increase both PWP and permeate flux, except for [62,205] due to the hydrophilization of the polymers and the enhancement of their antifouling resistance. Nevertheless, the regeneration is incomplete (*FRR* < 100% except in [208] for the membrane modified with WO_3_). Thus, chemical reagents are necessary for membrane cleaning; acids cause faster or slower degradation of photocatalysts. Polymers also degrade under the influence of both photocatalytic reactions and light [217]. Among various polymers, polytetrafluoroethylene, PVDF and PAN are the most chemically stable against these factors. However, PAN is cracked under light irradiation. Polydopamine is suggested to be an additional protector of PVDF against light [210].

Since photocatalytic filtration membranes are materials with strongly destructive action, they can be recommended for water and wastewater purification and disinfection, where permeate is a target product and must be free from organic additives as much as possible. When the solutions contain valuable organic components, photocatalysis can be used only for cleaning. Additionally, the usage of these membranes in tubular modules, especially in multichannel devices, is undesirable since uniform lighting is required.

## 8. Modifying 3D Printed Polymer Membranes

3D printing allows one to produce membranes of any shape and size according to the requirements of customers [218]. The methods of obtaining 3D-printed materials are considered “green”, since printing produces no solid wastes and requires no organic solvents or only a very small amount of them for ink preparation. For the fabrication of polymer-based membranes, the techniques of 3D inkjet printing [219,220,221,222,223,224], and fused deposition modeling [225,226] are used. Modification is performed by adding an inorganic constituent directly to the ink [219,220,222,224] or after printing the membranes (posttreatment procedure) [225,226].

As a rule, the membranes for oil–water separation have large voids (several hundred microns) [222,224,225,226]. The emulsion is separated under gravitational force by means of vacuum filtration; very high water fluxes (25,000 L m^−2^h^−1^ through the cellulose acetate/PVA/silica gel membrane [222], or even 40,000 L m^−2^h^−1^ through the GO-containing membrane [224]) are reported. An increase in the amount of silica gel nanoparticles enhances the membrane hydrophilicity and oleophobicity: a WCA and oil contact angle of 18° and 159°, respectively, were found [222]. At the same time, fumed silica provides strong hydrophobicity to polymers [226]. Regarding hydrophilic materials, the maximum retention of n-hexane (99.5%) [225], diesel, vegetable and lubricant oil (≈98%) [222] is reached for the membrane, whose pore size is 200 µm and less; the retention is independent of emulsion pH and salt concentration. High retention of non-polar liquids is explained as follows. The surface of printed polymer–inorganic materials is characterized by hierarchical rough structures. The surface acts as a trap for water, which prevents the penetration of hydrocarbon droplets through the membrane.

Poly(acrylonitrile-butadiene-styren) membranes were modified with nanoparticles of crystalline TiO_2_ [225]. No effect of inorganic nanoparticles on the pore size has been found, but they hydrophilize membranes. Relative to pristine material, modified membranes show a great reduction in WCA (from 95° to 15°); the retention of oil products exceeds 95%. GO was applied to modifying chitosan-based membranes; it increases the oil contact angle up to 161° [224]. Membranes were used for the separation of hydrocarbons and various oil products from water. The retention is the range of 35% (peanut oil) to 95% (n-heptane).

Membranes with ultrafiltration and antibacterial properties were fabricated in [222,223,224]. The effect of a co-solvent (dimethylacetamide) in the water–isopropanol ink on the morphology, functional properties, and performance of the chitosan/GO/AgNPs layer on glass, cotton, and polyester substrates was studied [219,220]. Increasing the co-solvent fraction and subsequently decreasing the chitosan content in the ink lead to a wider AgNPs distribution across the membrane surface [220]. Compared with the polyester substrate, the WCA for the 3D-printed membrane decreases from 55° down to 28°; the porosity shows a growth from 57% to 87%. The positive effect of the co-solvent is stressed; this is explained by its bonding with chitosan. The better performance of the membrane applied to the cotton surface has been found for the filtration of BSA solutions; the water permeability is at the level of 20 L m^−2^h^−1^ bar^−1^. The flux decreases due to fouling; however, the protein deposit can be easily removed (*FRR =* 81–97%). Chitosan/GO/AgNPs membranes were used for the inhibition of the strains of Gram-negative bacteria in surface water [219]. AgNPs were printed on a fibrous polyurethane membrane (the content of the inorganic constituent was even 69%), this material also depresses the development of Gram-negative bacteria [221].

It should be stressed that modifying 3D-printed membranes is insufficiently studied since this technique for the membrane production has been recently developed.

## 9. Advantages and Disadvantages of Inorganic Modifiers and Unresolved Problems of Modified Membranes

The literature provides a large amount of information about the morphology, functional properties and performance of polymer MF and UF membranes modified with inorganic nanoparticles. As shown in previous sections, all inorganic modifiers improve the performance of polymer membranes; particularly the enhancement of antifouling resistance is stressed in practically in all works. The advantages and disadvantages of common inorganic modifiers are summarized in Table 11. The advisability of the use of one or another inorganic substance is determined not only by good membrane performance, but also by the availability of the modifier, its cost, lifetime and safety for the environment and human health.

The analysis of the literature data allows us to note a significant lack of information. A strictly scientific problem is the influence of embedded inorganic particles on the polymer constituent, since interesting results could be discovered. For instance, AgNPs transform PVDF into the polar β phase; this increases the surface charge of the modified polymer enhancing its antifouling resistance [106]. As a rule, such transformations of one or another polymer, which are affected by inorganic particles, are not studied. The effect of the size of primary particles on the functional properties of filtration membranes is also outside attention. Slower or faster gradual degradation of membrane performance is noted in many works, but its reason remains unclear. It can be caused by the fouling of the polymer, adsorption of organic species on the modifier particles, or their degradation or leakage. Although the effect of the modifier amount on the hydrophilicity, separation ability and antifouling resistance of polymer membranes is considered in detail, the required content of the inorganic constituent is found empirically, except probably [161]. Mathematical modeling is desirable to evaluate the optimal composition of modified membranes. From a practical point of view, a very serious problem is the leakage of inorganic particles (especially those located on the membrane surface) caused by flow. As a rule, the chemical analysis of membranes after pure water filtration is not performed despite the importance of the problem, since the release of certain modifiers into the environment must be avoided due to their toxicity.

In the case of AgNPs, the leakage of Ag^+^ ions, which are generated due to silver corrosion, is usually studied. The testing duration is up to 30 days. However, attention is not focused on the stability of the nanoparticles themselves in polymers. It is important since corrosion is accompanied by the reorganization of AgNPs: larger nanoparticles are broken into smaller ones, which can be released more easily [111]. Moreover, the long-time resistance of the membranes against biofouling and also the chemical stability of polymers against ROS produced due to bacterial degradation are not investigated. Mainly antibacterial activity of as-prepared membranes is studied. Moreover, attention is not focused on the cause of the hydrophilizing ability of AgNPs, since adsorbed reducing agents and reduction products can be removed from them during polymer modification and membrane operation.

Another problem is the possibility of modifier degradation in acidic media, as is typical, for instance, for metal oxides. As a rule, the *FRR* parameter after each cycle of solution filtration–regeneration with water is less than 100% indicating the incomplete removal of fouling from the membranes. This means that reagents must be used sooner or later. However, tests for the estimation of the long-time reusability of modified membranes are not conducted. The studies are limited to several cycles.

Regarding photocatalytic membranes, the problem is to make them more sensitive to visible light. Thus, the search for efficient, chemically stable photocatalysts is an actual task. Very promising materials are self-cleaning membranes; they can be removed from modules after filtration and irradiated with light. In this case, special transparent modules are not required. The polymer must be stable against both ROS and light; fluorine-containing polymers are the most promising.

Modifying 3D-printed membranes is complicated by the absence of information about the optimal composition of the ink, the interaction of modifiers with polymers at elevated temperatures, the transformation of the functional properties of modifiers during printing, and the effect of the printing conditions on the morphology, properties and performance of the resulting membranes. Surface modification is applied mainly to grid-like separators. Regarding membranes containing pores of micron size and smaller, such investigations have not been performed. No attempts to fill such pores of 3D-printed polymer matrices are known.

Finally, the laboratory samples must be tested using not only model solutions (as in the majority of works) but also real fluids. The solution of all the mentioned practical problems is necessary for the industrial-scale production of membranes modified with inorganic modifiers.

## 10. Conclusions

In this review, modified materials based on the polymers that are traditionally used for the production of MF and UF membranes are considered. The effect of various inorganic substances on the morphology, functional properties, and performance of the modified membrane is analyzed. The modifiers increase the fluid permeability, enhance the retention of one or another species, improve mechanical properties, and reduce fouling with organic substances and biofouling. The improvement of the membrane performance is achieved due to the effect of inorganic nanoparticles on the porous structure of polymers, their hydrophilicity and surface charge. The fluid permeability is often several times higher than that for the unmodified polymer; it can even exceed 1000 L m^−2^h^−1^bar^−1^. However, sometimes such high values are achieved for membranes whose polymer base itself exhibits considerable permeability. The same can also be attributed to retention ability. For instance, the modified membrane retains species practically completely, whereas the retention is 95% for the pristine polymer. Nevertheless, this small growth is compensated by a significant improvement in other membrane characteristics. Sometimes the fluid permeability decreases compared with the pristine polymer, but the modified membranes become capable of retaining smaller species. Higher antifouling resistance of the membranes containing inorganic modifiers is stressed in all cases. Functionalized modifiers and their composites open additional perspectives: the membranes containing them show better performance than the materials modified with unfunctionalized particles or single-component modifiers.

From an economic point of view, the most advantageous option is to use aluminosilicates, oxides or phosphates of multivalent metals as well as CDs, since their nanoparticles can be obtained via simple synthesis procedures from available and relatively cheap (even low-cost) feedstock. Regarding the membranes containing more expensive modifiers, for instance, GO, AgNPs, MXenes or photocatalysts doped with noble metals, the optimal modifier content is usually less than 1%. This means that the modifier contribution to the membrane cost cannot be very significant. Nevertheless, the higher cost of modified membranes can be compensated by the lower energy and material costs of the separation processes (no increase in pressure due to fouling, lower consumption of reagents and pure water for regeneration and rinsing the membrane systems).

From the environmental point of view, CDs and zeolites are synthesized with a “green” hydrothermal technique, AgNPs can be obtained using plant extracts, and GO can be reduced hydrothermally or using environmentally friendly agents. Among the modifying methods, some approaches to surface engineering can be considered as “green”; for instance, layer-by-layer assembly using tannic acid. Filling the pores of pre-formed polymer membranes using aqueous solutions is also more or less environmentally friendly. Among the modified materials, photocatalytic membranes look the most attractive due to their self-cleaning ability and the possibility of complete degradation of toxic contaminants (no hazardous waste). However, the separation enhanced by photocatalysts cannot be applied to biotechnology and the food industry due to the degradation of target products. Nevertheless, contaminated membranes can be cleaned with light irradiation.

The directions of further investigations involve, first of all, a search for new efficient and chemically stable inorganic modifiers (for instance, heterojunction structures, functionalized nanomaterials, nanocomposites), and also a decrease in the cost of known modifiers. Such scientific problems as the size effect of embedded primary particles on the membrane properties, the reasons for the degradation of membrane performance, etc., still await a solution. A number of practical problems have to be solved, for instance, the application of modified membranes to various objects, especially to the real solutions of natural or technogenic origin. Further investigations should also involve the long-time leakage and degradation of the modifiers to evaluate the lifetime of membranes and their harmlessness to the environment and human health. The preparation of composite membranes involving 3D printing also requires the solution of a number of problems (the optimal ink composition and printing conditions, the choice of a modifier to provide the best membrane performance etc.). All this information is necessary for the large-scale industrial production of modified membranes. Since the efficiency of all modifiers is reported, the usage of one or another known inorganic substance is determined by its cost, availability, multifunctionality and chemical stability, and also by the simplicity and environmental safety of the modifying procedure.

Currently, the optimal membrane composition and modifying conditions are found empirically. An actual task is the development of a unified theoretical approach in order to obtain materials with the best functional properties. The models would take into consideration the types of polymers and modifiers, their ratio and the modifying conditions. This approach would be very useful, since modified membranes are promising for practical applications, especially in combination with various antifouling strategies.

## Figures and Tables

**Figure 1:**
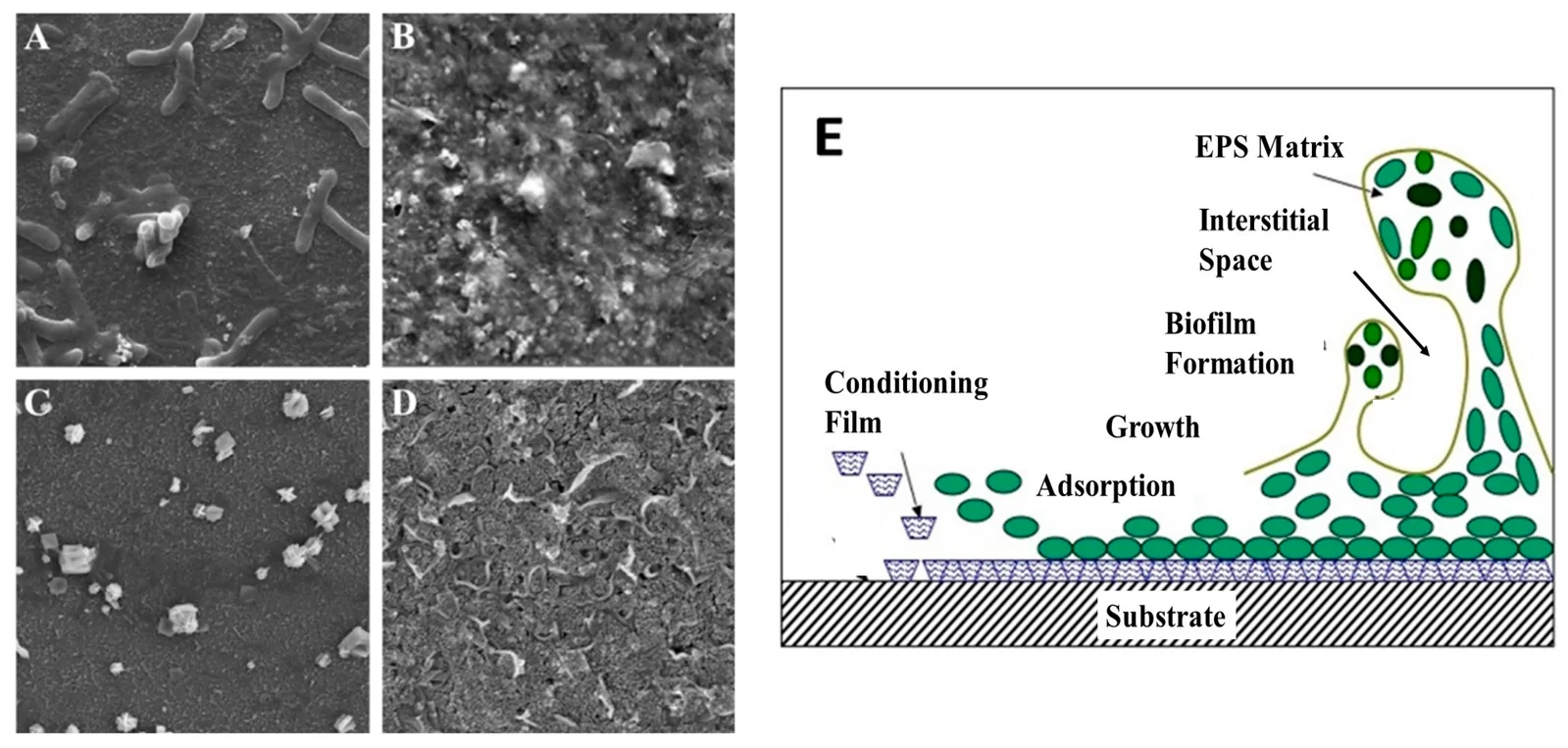
SEM images of: (**A**) biofouling; (**B**) organic fouling; (**C**) inorganic fouling; (**D**) colloidal fouling [50], (**E**) evolution of biofouling [51]. Reproduced with the permission from Elsevier (**A**–**D**) and Springer (**E**).

**Figure 2:**
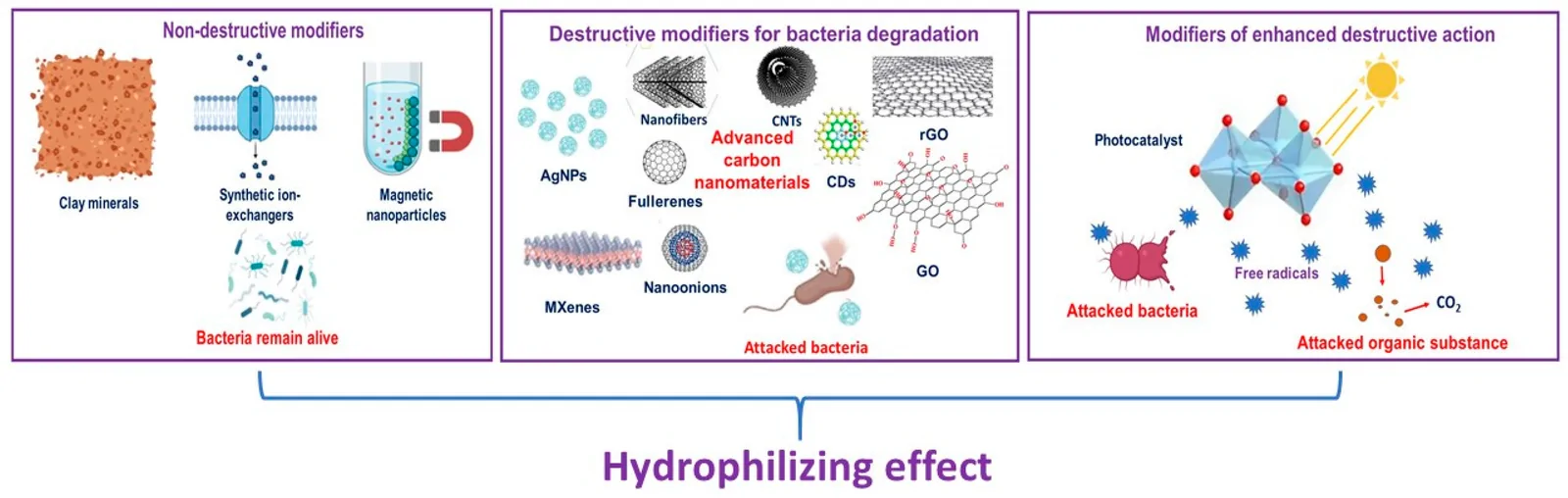
Classification of inorganic modifiers and their effect on bacteria and organic substances.

**Figure 3:**
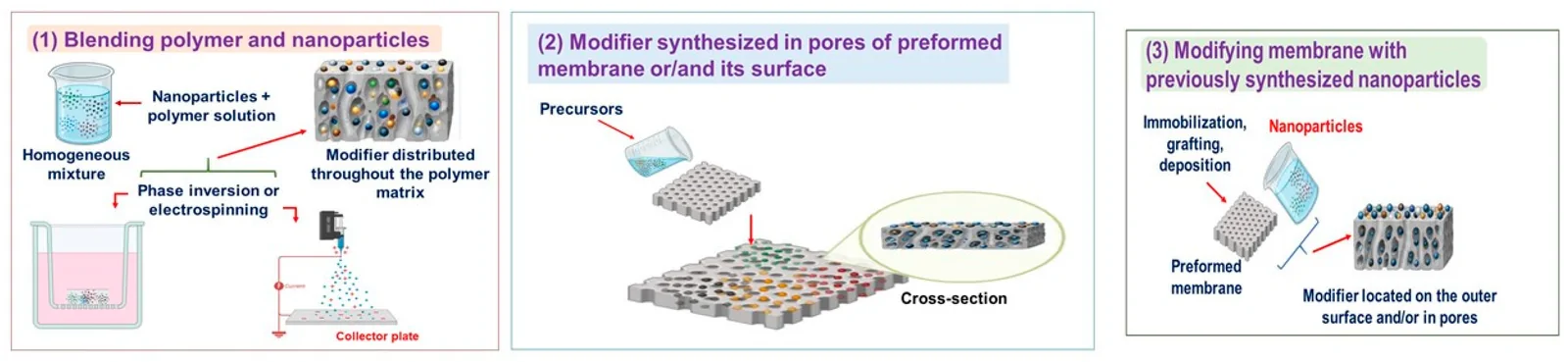
Modifying porous filtration membranes.

**Figure 4:**
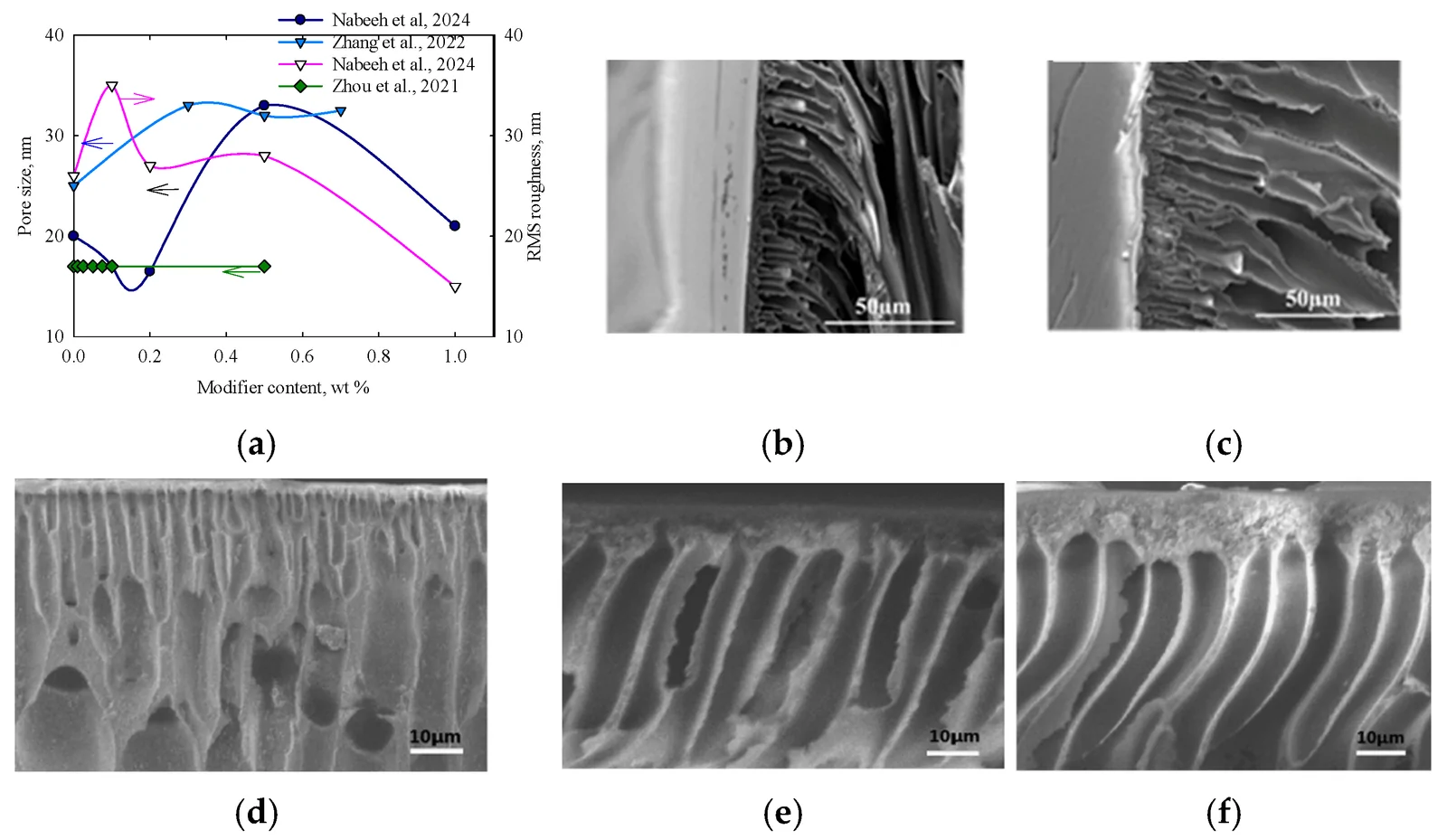
Effect of the modifier content on (**a**) the average pore size and surface roughness, and (**b**–**f**) pore shape of polymer-based membranes observed from the SEM image. Polymers: (**a**) polysulfate [73]; (**a**–**f**) PES [74,78,79] and modifiers: (**a**) MXenes [73,75], CDs [74], (**c**) carbon nano-onions [78], (**d**–**f**) functionalized GO [79], modifier content (wt%): ((**b**) [78], (**d**) [79]) 0, ((**c**) [78], (**e**) [79]) 1, ((**f**) [79]) 3. (**a**) Plotted according to data [73,74,75], (**b**–**f**) reproduced with the permission from Elsevier.

**Figure 5:**
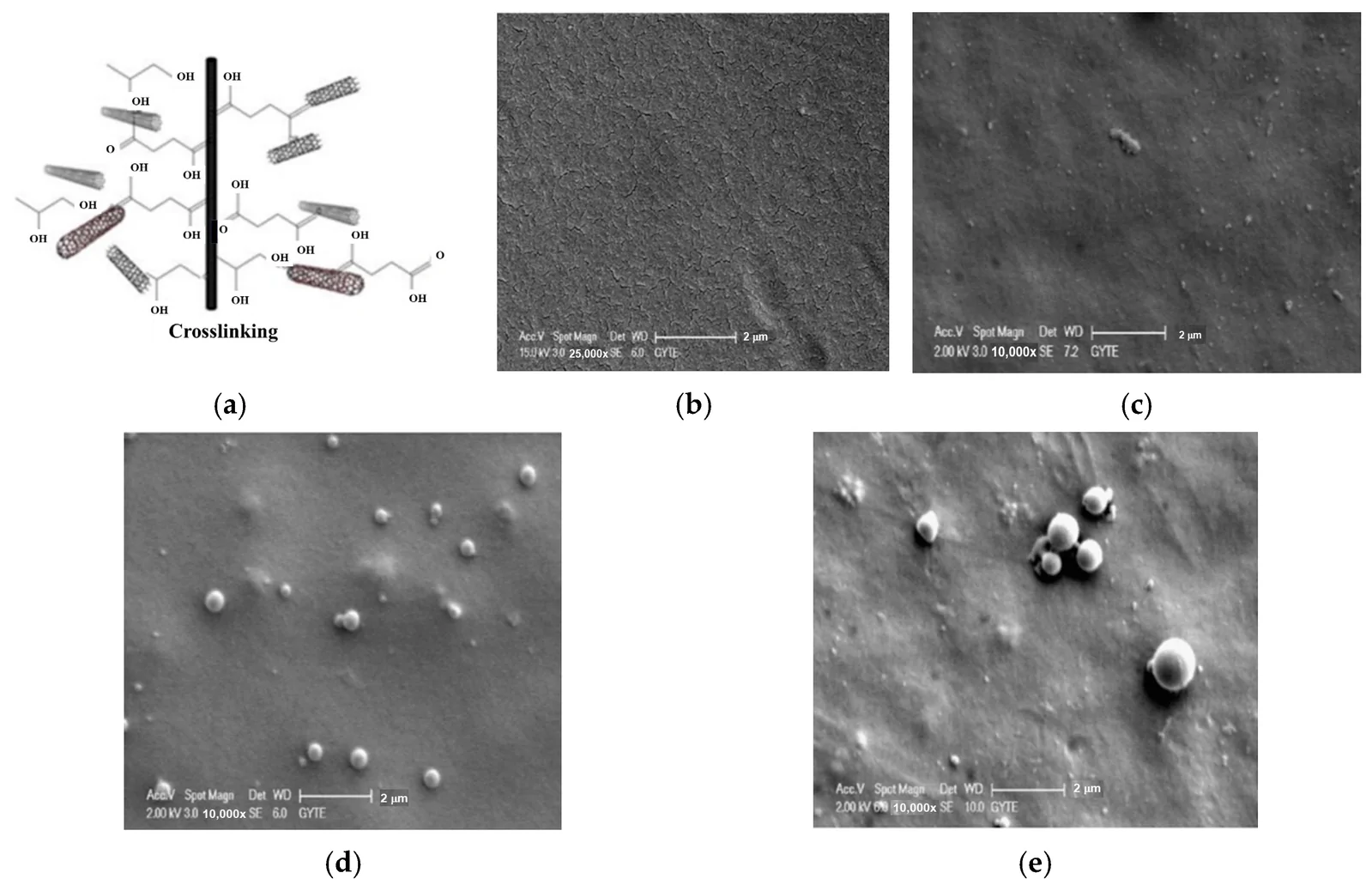
(**a**) Cross-linking of polymer (in the example of CNTs) [81], (**c**–**e**) SEM images of AgNPs on the surface of PS membrane [82], (**b**–**e**) the content of modifier (wt%): (**b**) 0, (**c**) 0.25, (**d**) 0.5 (**c**), (**e**) 1 [82]. Reproduced with the permission from Elsevier.

**Figure 6:**
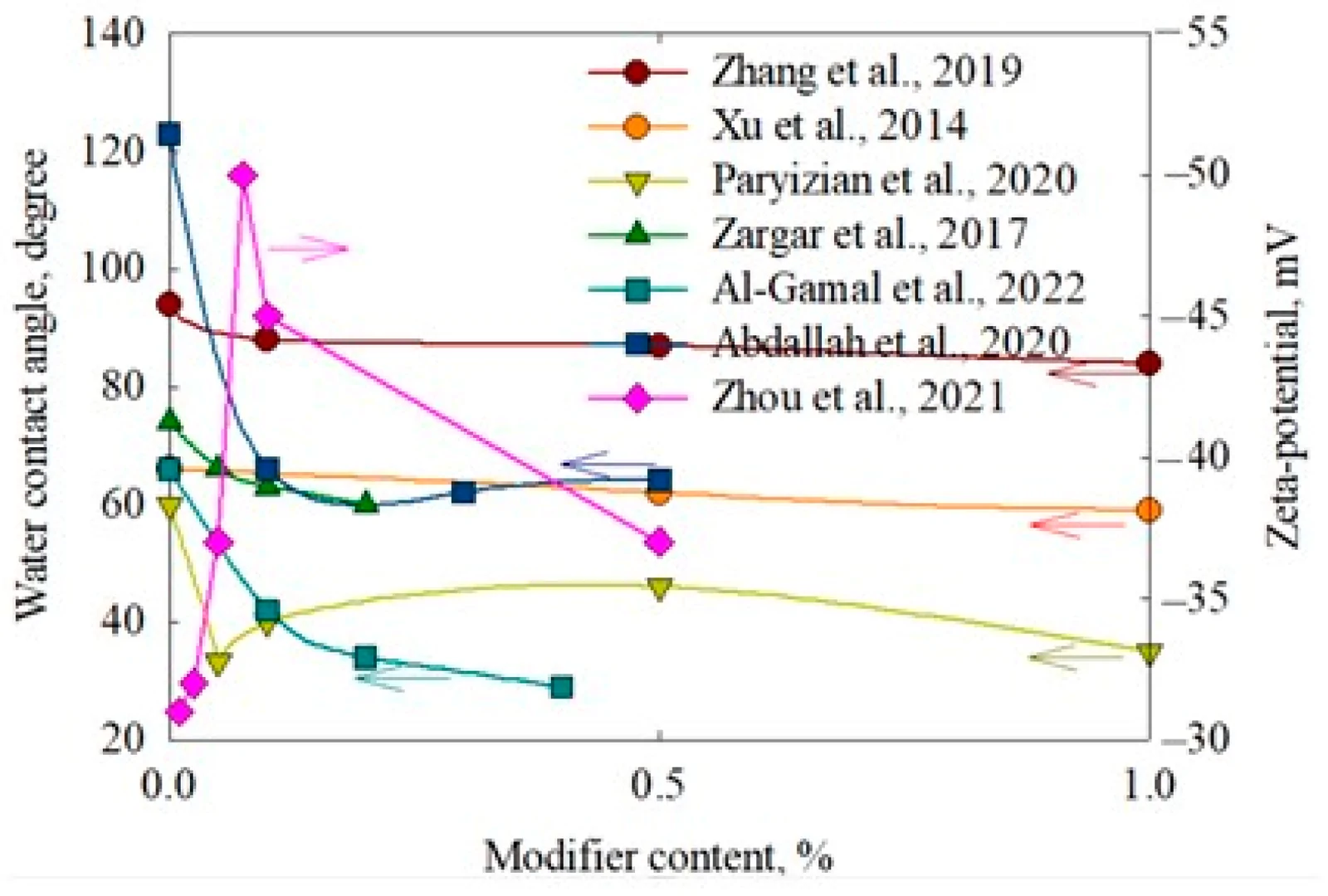
WCA and zeta-potential as functions of the modifier content in polymers, plotted based on data from [73,85,86,87,88,89,90]. Modifiers include: MXene [73], functionalized silica [85,86,87], functionalized TiO_2_ [88], GO/functionalized silica [89], and functionalized GO [90].

**Figure 7:**
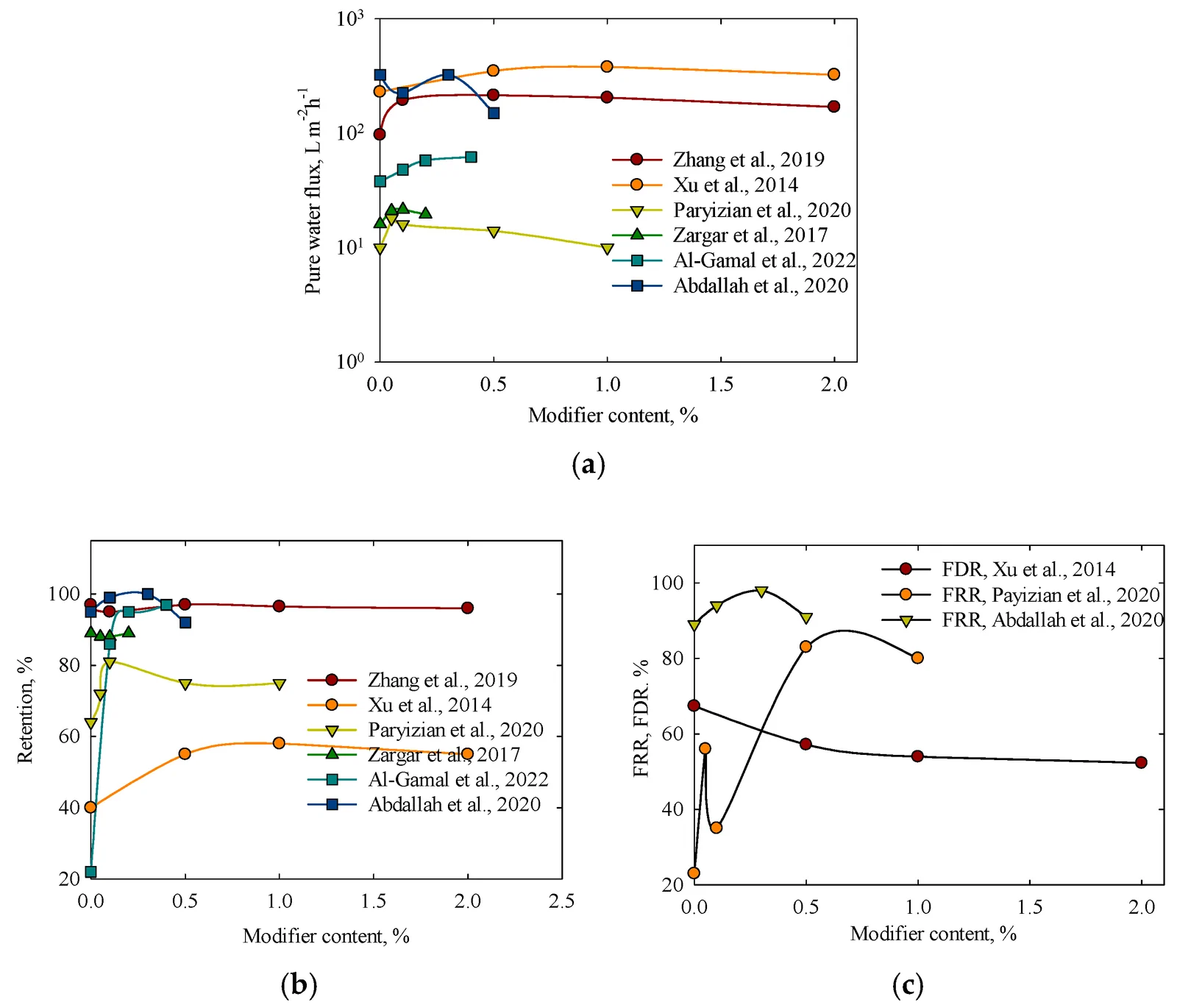
(**a**) Pure water flux; (**b**) retention of species; (**c**) fouling parameters as functions of modifier content in polymers, plotted based on data from [85,86,87,88,89,90]. Modifiers: functionalized silica [85,86,87], functionalized TiO_2_ [88], composite of GO/functionalized silica [89], functionalized GO [90]. Species: BSA [85,89,90], Ca and Mg salts [86], NaCl [87], Na_2_SO_4_ [88].

**Figure 8:**
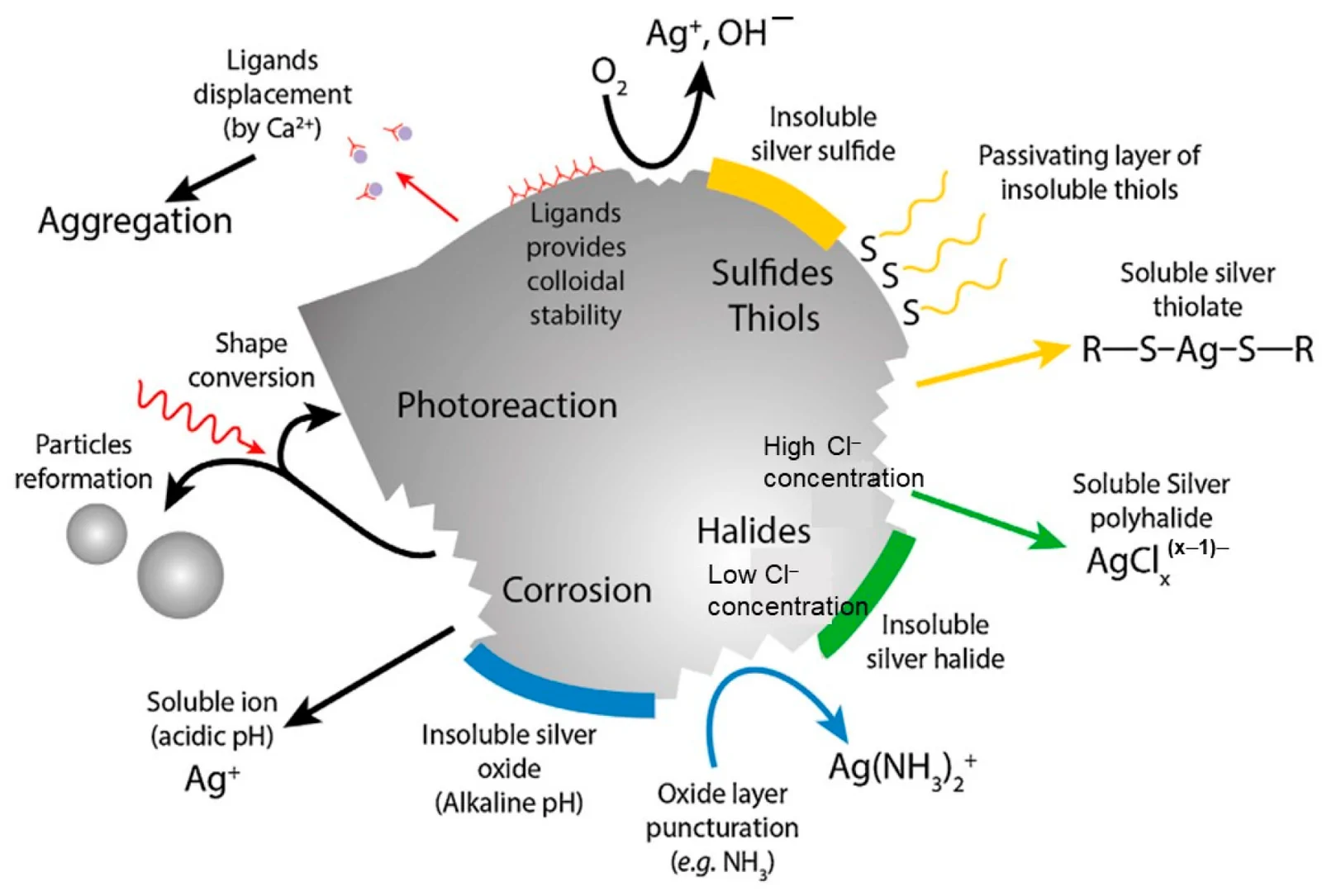
Degradation of AgNPs in various media [112]. Reproduced with the permission from Elsevier.

**Figure 9:**
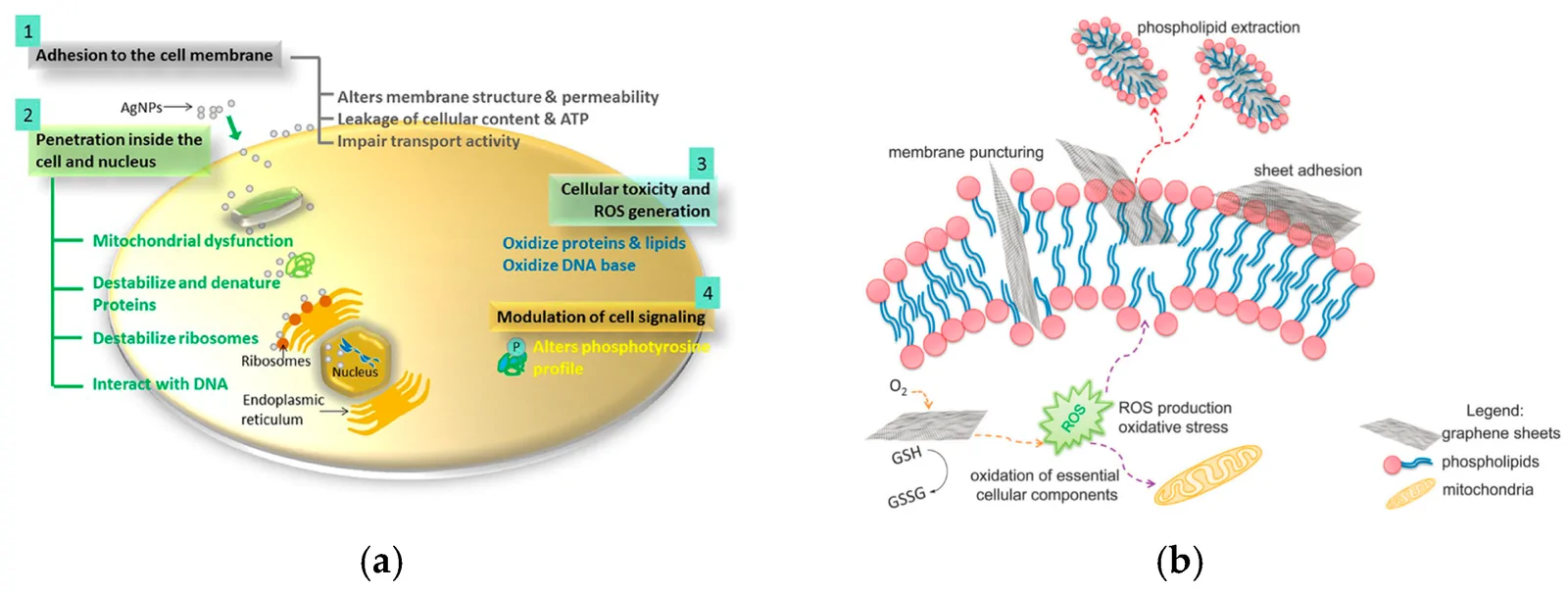
Cell damaging affected by (**a**) AgNPs and (**b**) GO nanosheets [44]. Reproduced with the permission from Elsevier.

**Figure 10:**
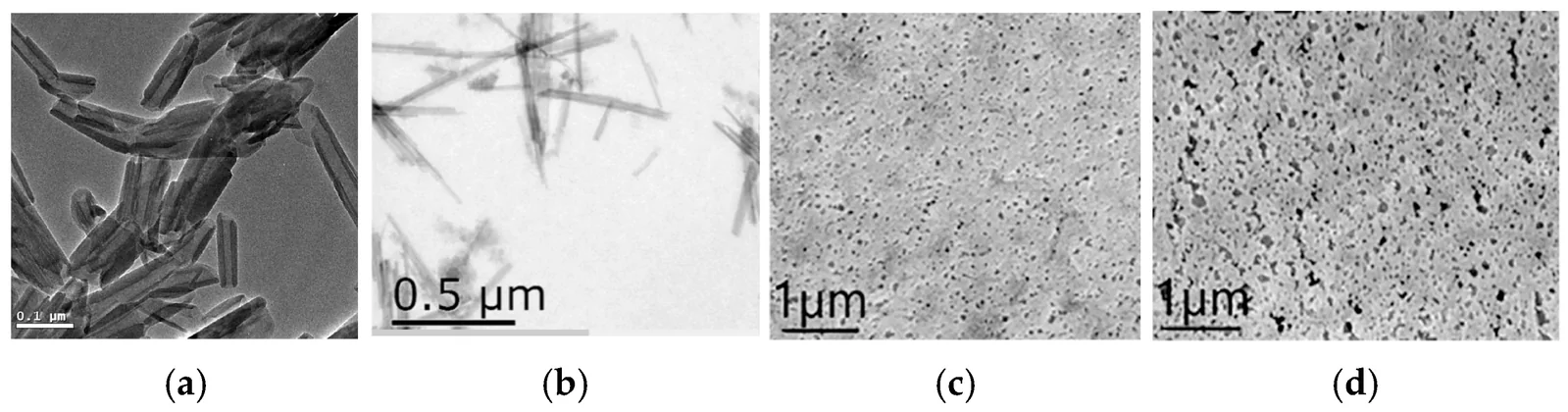
(**a**) Halloysite [91] and (**b**) palygorskite nanorods; (**c**) the outer surface of pristine and (**d**) palygorskite-containing PVDF membrane [92]. Reproduced with the permission from Elsevier.

**Figure 11:**
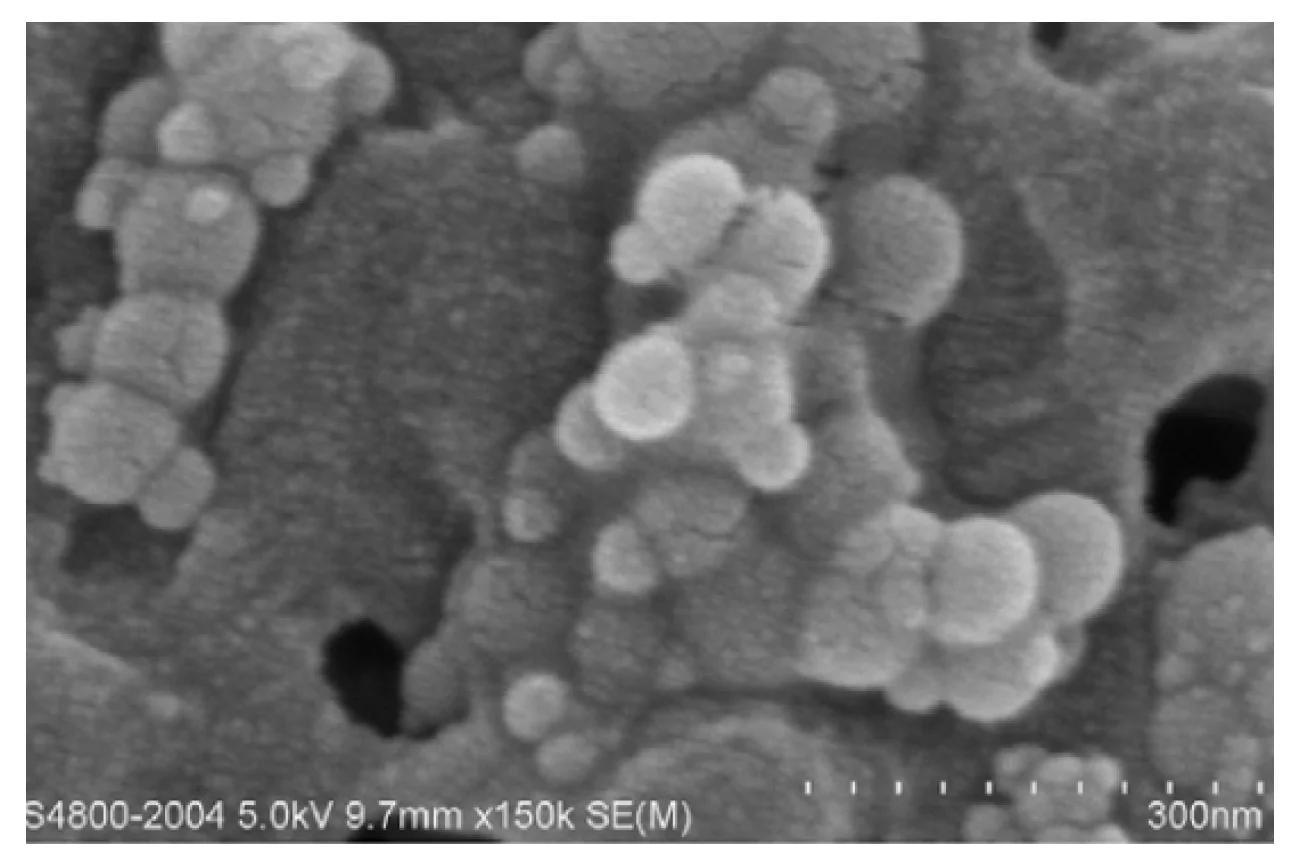
Nanoparticles of hydrated aluminum oxide on the surface of PVDF membrane [135]. Reproduced with the permission from Elsevier.

**Figure 12:**
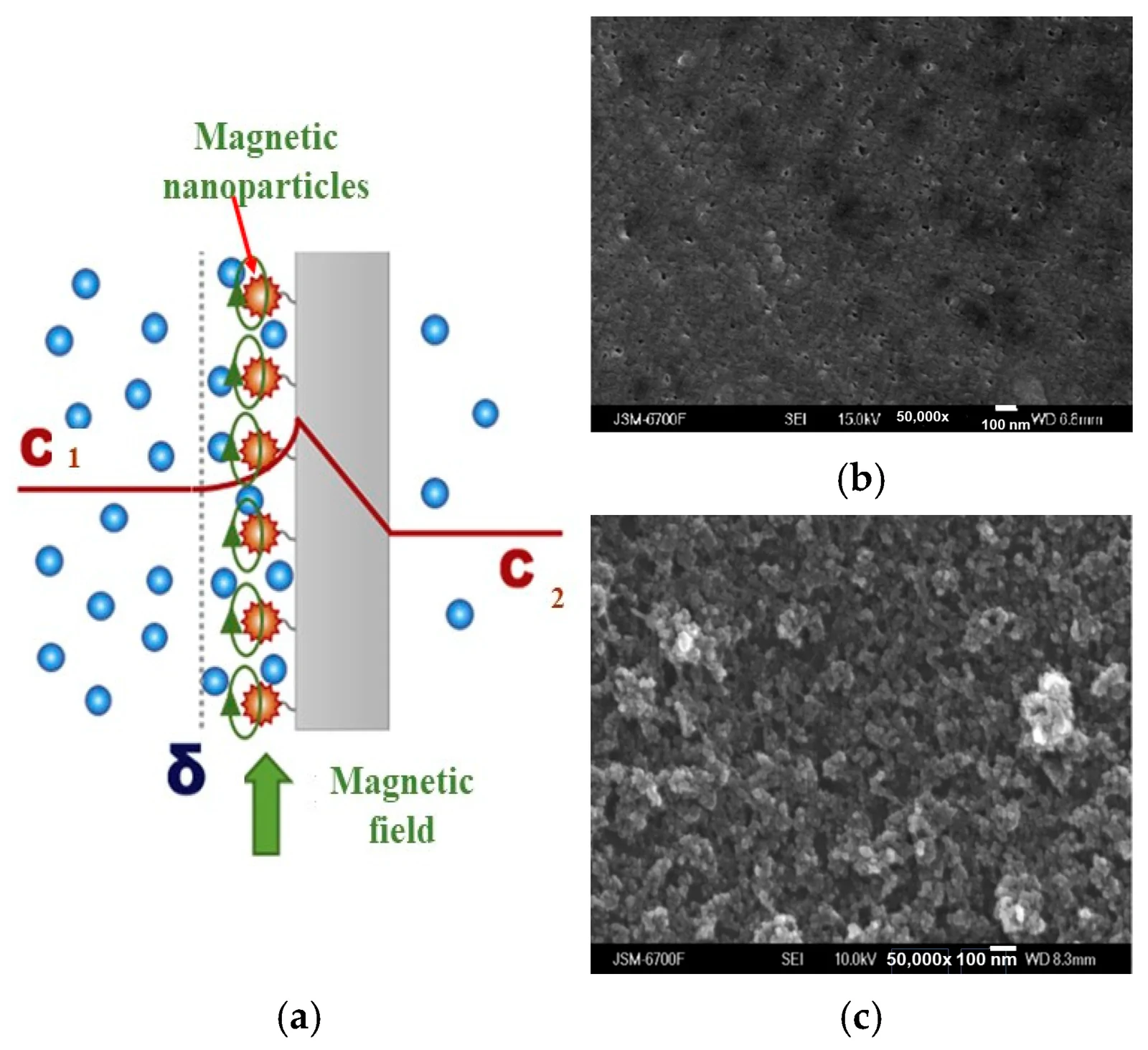
(**a**) Scheme of the membrane surface modified with magnetic nanoparticles; SEM images of (**b**) pristine PS and (**c**) modified membranes [47]. Reproduced with the permission from Elsevier.

**Figure 13:**
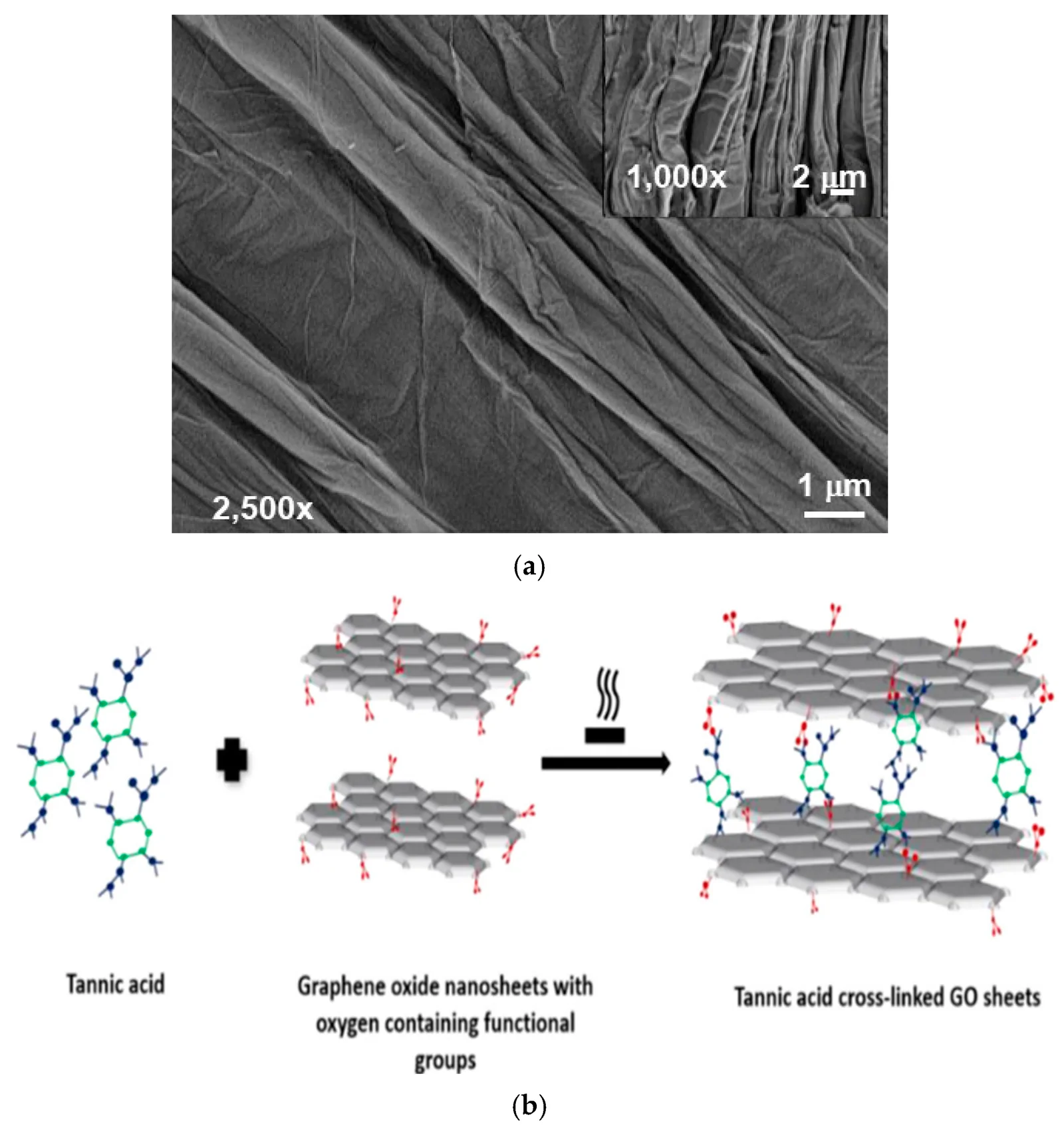
(**a**) GO flakes on the membrane surface; (**b**) formation of the GO/tannic acid composite [153]. Reproduced with the permission from Elsevier.

**Figure 14:**
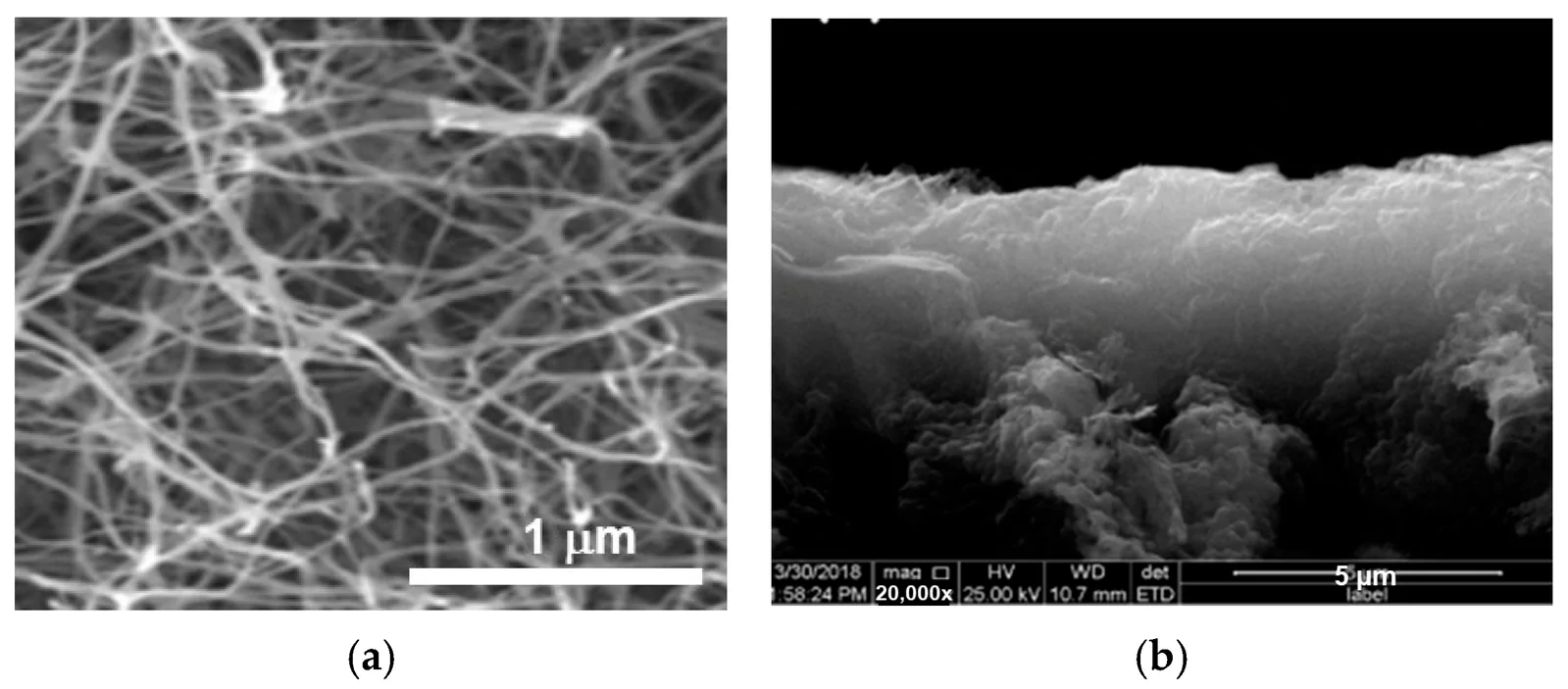
SEM images of (**a**) carbon aerogel and (**b**) membrane surface modified with aerogel [175]. Reproduced with the permission from Elsevier.

**Figure 15:**
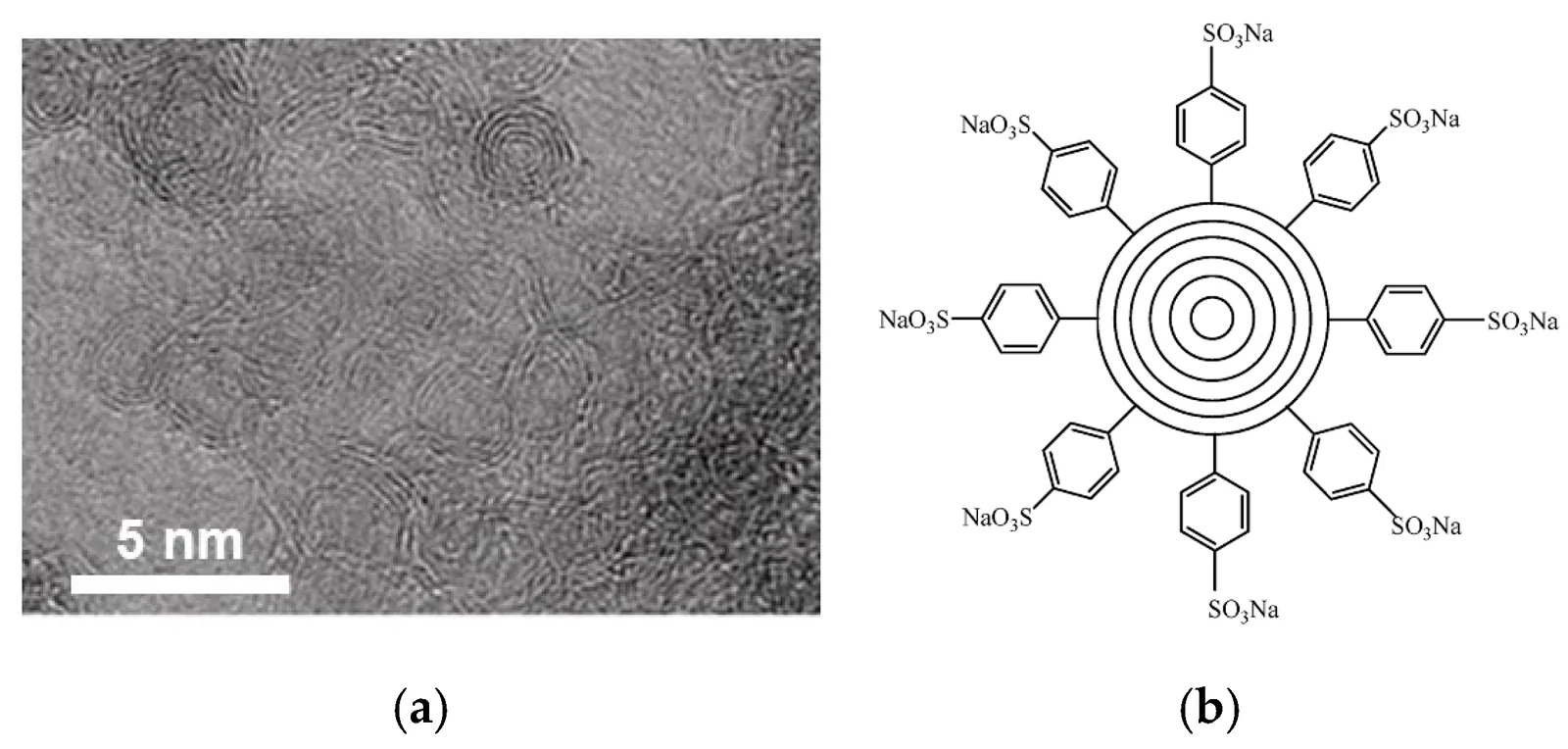
(**a**) TEM image of nano-onions; (**b**) nano-onions functionalized with sulfophenol groups [78]. Reproduced with the permission from Elsevier.

**Figure 16:**
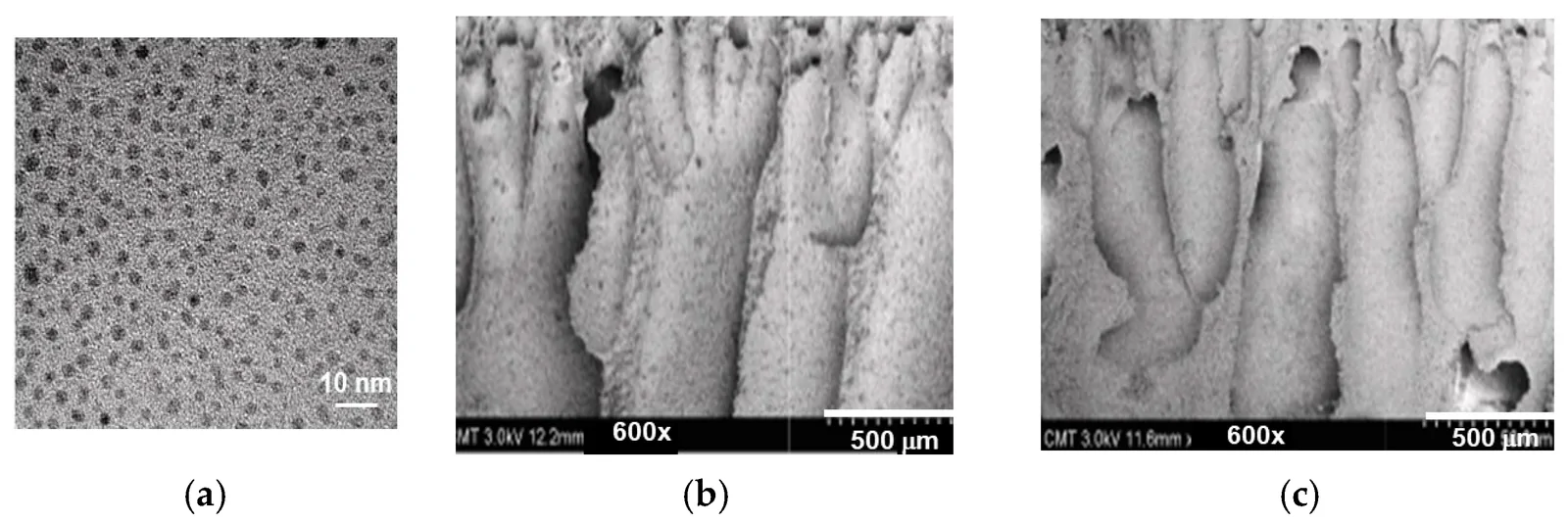
SEM images of (**a**) CDs, (**b**) cross-sections of pristine membrane and (**c**) membrane containing CDs functionalized with zwitterions [180]. Reproduced with the permission from Elsevier.

**Figure 18:**
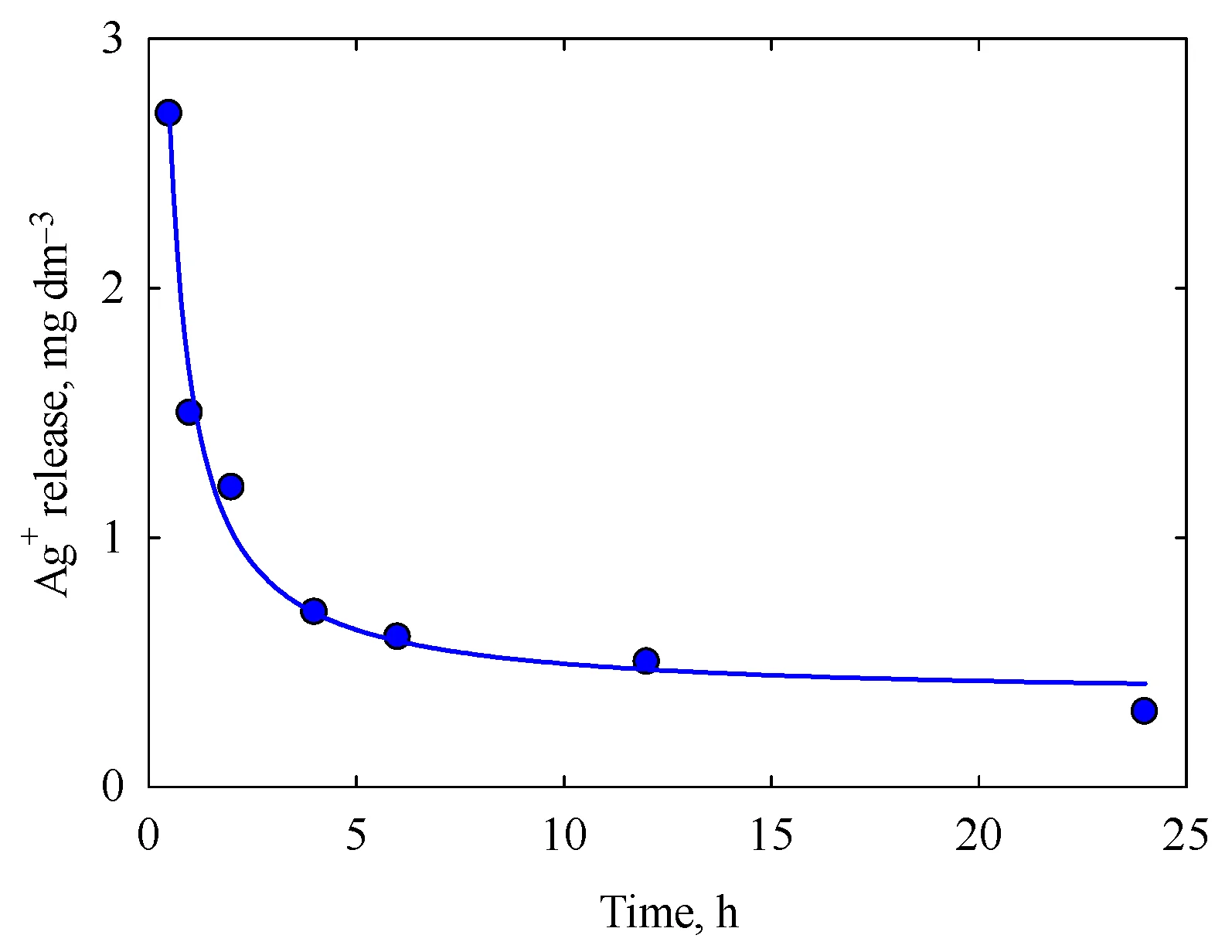
Release of Ag^+^ ions from the PS membrane modified with AgNPs, plotted based on [199].

**Figure 19:**
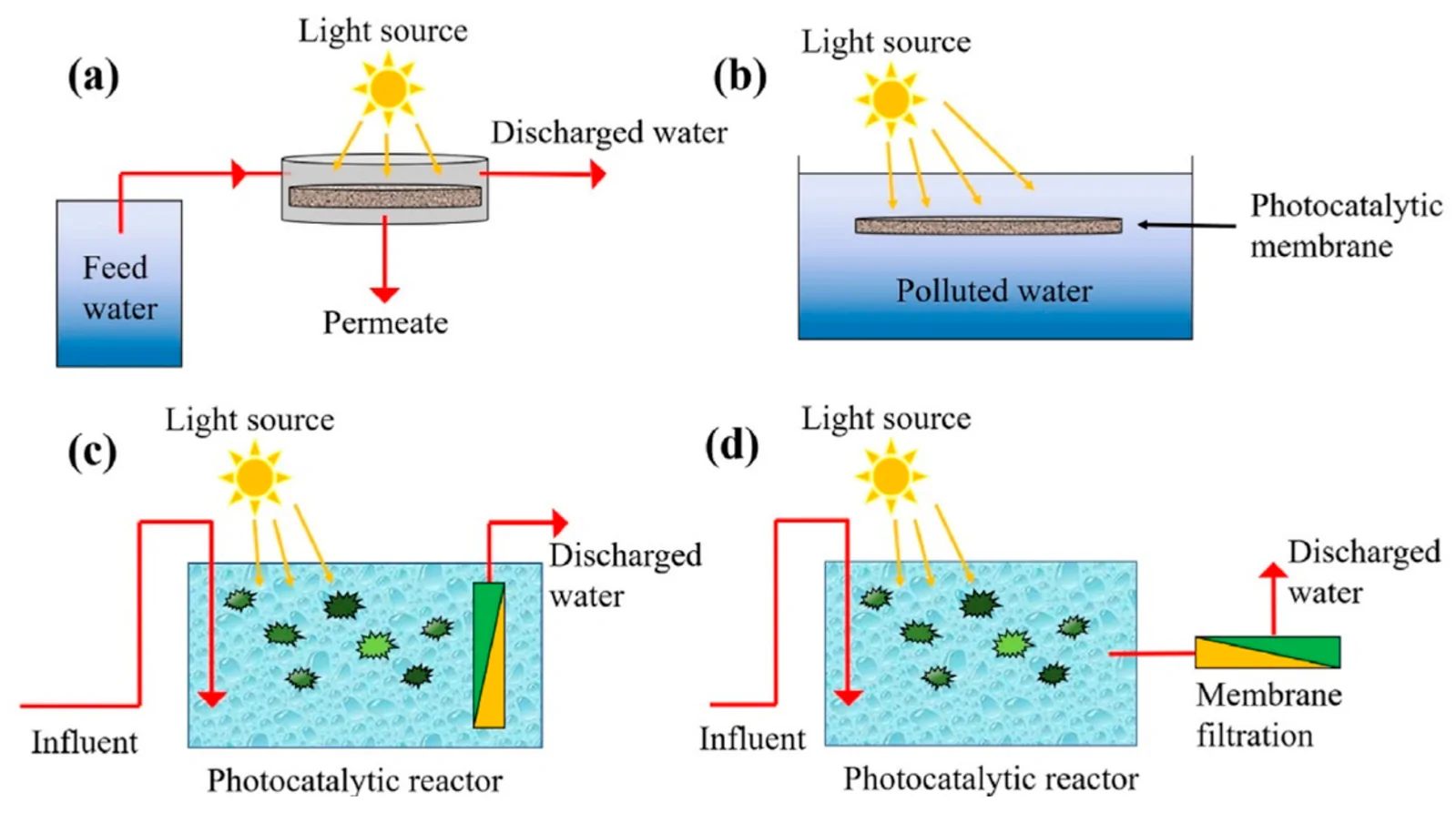
Photocatalytic systems: (**a**) filtration of the solution through a photocatalytic membrane; (**b**) immersion of a photocatalytic membrane in a liquid; (**c**) photocatalyst particles suspended in a liquid, which fills the module with a conventional membrane; (**d**) independent photocatalysis and membrane filtration units [202]. Reproduced with the permission from Elsevier.

**Table 1:** Effect of modifiers on mechanical properties of polymer membranes.

Polymer	Modifier	Tensile Strength (MPa)	Elongation at Break (%)	Young’s Modulus (MPa)	Elastic Strain (%)	Hardness (MPa)	Ref.
PVDF	-	6	130	65	-	-	[99]
	zeolite	3.5	25	120	-	-	
	zeolite/silica-thiol groups	6	35	180	-	-	
Polyurethane	-	1.5	305	2	-	-	[98]
	laponite	6.5	200	35	-	-	
	GO	6	90	15	-	-	
	laponite/GO	17	105	65	-	-	
PS	gelatin	-	-	450	-	55	[101]
	gelatin/Zr phosphate	-	-	800	-	85	
Poly-(m-phenyleneisophalamide	-	3	40	-	-	-	[30]
	TiO_2_	5	65	-	-	-	
PS	-	-	-	-	4.2		[104]
	ZrO_2_	-	-	-	2.6		
PES	-	3.8	13	-	-	-	[105]
	GO/TiO_2_	4.5	24	-	-	-	
PVDF	-	40	18	-	-	-	[85]
	silica-aminogroups	85	30	-	-	-	
PVDF	-	1.4	8	-	-	-	[89]
	GO	1.3	7.5	-	-	-	
	GO/silica-aminogroups	2.4	11	-	-	-	
PS	-	3.5	10	131	-	-	[45]
	GO	6.3	12	181	-	-	
PES	-	2.7	14	59	2	-	[79]
	GO/PEI	3.5	19	72	4	-	
PVDF	-	3	55	-	-	-	[29]
	AgNPs	5	90	-	-	-	
	GO/AgNPs	11	100	-	-	-	
PVDF	-	1.5	30	-	-	-	[106]
	AgNPs	2	70	-	-	-	
-	Ti_3_C_2_T_x_	30	-	1	15	-	[96]
PVA	-	22	-	3.5	1	-	
	Ti_3_C_2_T_x_	91	-	3.7	4	-	

**Table 2:** Functional properties and performance of membranes modified with aluminosilicates.

Polymer	Modifier	WCA (Degree)	Filtration				Ref.
			PWP (L m^−2^h^−1^bar^−1^)	Solution Component	Retention (%)	*FRR* (%)	
PES	-	90	80	PVA (30–70 kDa)	100	86	[91]
	Halloysite/Dextran	58	225		95	96	
PVDF	-	96	160	BSA	100	52	[92]
	Palygorskite	84	280		99.4	80	
PVDF	-	155	-	dye, surfactant, oil, microplastic	25–99	-	[124]
	Natural zeolite	130	-		80–99	-	
PVDF	-	77	18	dye	87	87	[122]
	Natural zeolite—Surfactant	65	7		98	96	
PES	-	65	-	Pb(NO_3_)_2_	28	-	[76]
	Synthetic zeolite	50	-		97	-	
PVDF	-	75	2	pepsin, BSA	82–95		[123]
	Synthetic zeolite	67	3		92–99		
PVDF	-	64	12	Methylene Blue	82	84	[99]
	Zeolite	65	9		97	80	
	Zeolite/silica-thiol groups	67	16		97	88	
PAM	-	65	2	Humic acids	90	-	[100]
	Bentonite	60	2		95		
	Bentonite/silica-thiol groups	55	12		98		

**Table 3:** Functional properties and performance of membranes modified with phosphates and oxides of multivalent elements.

Polymer	Modifier	WCA (Degree)	Filtration				Ref.
			PWP (L m^−2^h^−1^bar^−1^)	Solution Component	Retention (%)	*FRR* (%)	
PSF-PAM	-	75	420	Na_2_SO_4_	-	-	[101]
	Gelatine/zirconium hydrophospate	-	17		95	94	
PVDF	-	123	-	Humic acids, BSA	≈95	83–89	[85]
	Silica—aminogroups	77	-		>99	98–99	
PAM	-	66	-	CaCl_2_, MgSO_4_, Pb(NO_3_)_2_, CdSO_4_, Ni(NO_3_)_2_, Cr(NO_3_)_3_	-	-	[86]
	Silica	50	3.5		16–35		
	Silica-aminogroups	29	6.2		>99		
PES+PAM	-	75	≈1	NaCl	90	-	[87]
	Silica	60			88		
	Silica-aminogroups	45			93		
PS	-	65	40	BSA	99	87	[137]
	Silica	54	45		97	90	
	Silica-PEI	51	70		99	95	
PS	ZrO_2_	-	68	-	-	-	[104]
PAM		69	324	Pesticides	0	-	[19]
	ZrO_2_	43	108		90	-	
PAN	-	73	20		97	-	
	ZrO_2_	65	3		98	-	
PVDF	-	92	1080		0	-	
	ZrO_2_	76	612		96	-	
PES	-	70	370	Dextran, 500 kDa	98	60	[134]
	TiO_2_ (rutile)	60	350		97	57	
	TiO_2_/silica-aminogroups	63	470		92	84	
PES	-	78	2	Cu(NO_3_)_2_	15	-	[97]
	Fe_3_O_4_	72	5		35	-	
	Fe_3_O_4_/silica	67	8		35	-	
	Fe_3_O_4_/silica-aminogroups	75	4		80	-	
PVDF	-	68	45	Proteins	-	63	[138]
	Magnetic Fe_3_O_4_ nanoparticles	50	75		69–90	71	

**Table 4:** Functional properties and performance of GO-containing membranes.

Polymer	Modifier	WCA (Degree)	Filtration					Ref.
			PWP (L m^−2^h^−1^bar^−1^)	Solution				
				Component	Retention (%)	*FDR* (%)	*FRR* (%)	
PVDF	-	79	-	yest	-	-	52	[161]
	GO ^1^	60	-		-	-	81	
PS	-	66	34	Pb(NO_3_)_2_	93	26	-	[45]
	GO ^1^	34	54		95	74	-	
PES	GO/PEI ^2^	59	99	dye	98	64	85	[94]
PES	GO/chitosan ^2^	64	50	dye	100	80	75	[154]
PES	GO/PVA ^2^	61	14	dye	>99	57	80	[95]
PES	GO/TA ^2^	87	17	dyes	90–100	41	67–87	[63]
PES	GO/TA ^2^	92	19	dyes	100		71	[158]
Polyurethane	-	93	5	dyes, Na_2_AsHO_4_ Pb(NO_3_)_2_	5–27	-	-	[98]
	GO/laponite ^1^	70	200		86–98	-	-	

^1^ Modifier is distributed in polymer; ^2^ Modifier is located on surface.

**Table 5:** Functional properties and performance of membranes coated with rGO.

Polymer	Modifier	WCA, Degree	Filtration				Ref.
			PWP, (L m^−2^h^−1^bar^−1^)	Solution			
				Component	Permeability (L m^−2^h^−1^bar^−1^)	Retention (%)	
PVDF	GO	67	-	dye	5	96	[165]
	rGO	81	-		30	>99	
Acetylcellulose	rGO	26	13	dye	-	72	[167]
PVDF	GO	55	30	dyes	28	99	[168]
	rGO	65	37		25	95–100	
PVDF	GO	62	20	PAC	9–15	75–78	[169]
	rGO	104	1024		600–≤1000	50–55	
PAM	rGO-MOF	42	22	dyes Cu(NO_3_)_2_ Cd(NO_3_)_2_	8–23 5 22	45–99 65 93	[166]

**Table 6:** Functional properties and performance of polymer membranes modified with 1D nanomaterials.

Polymer	Modifier	WCA (Degree)	Filtration				Ref.
			PWP (L m^−2^h^−1^bar^−1^)	Solution Components	Permeability (L m^−2^h^−1^bar^−1^)	Retention (%)	
PES	-	86	-	CuCl_2_, FeCl_3_, NiCl_2_, ZnCl_2_	25	50–100	[171]
	oxidized CNTs	73	-		50	75–100	
PAN	oxidized CNTs	39	5800	COD of surface water	4500	30	[81]
PS	-	54	500	dye	100	20	[174]
	oxidized and aminated CNTs	45	-		70	78	
PS	chitosan	73	1	Cu(NO_3_)_2_, Ni(NO_3_)_2_, Pb(NO_3_)_2_, Cd(NO_3_)_2_, Ba(NO_3_)_2_, Co(NO_3_)_2_,	-	-	[170]
	CNTs/chitosan	63	2		4	70–90	
	oxidized CNTs/chitosan	59	4		6	70–90	
PES	-	73	150	BSA, humic acids, Na alginate, yeast	30–270	45–80	[172]
	CNTs	65	650		100–420	62–82	
	CNTs-Ni	52	1100		100–750	90–100	
Chitosan	CNTs	70	81	dye	81	85	[173]
	CNTs/Iodine	60	220		220	65	
PVDF	-	-	-	BSA, Na alginate	80–140	55–92	[175]
	Carbon nanofibers	-	-		195	95–97	

**Table 7:** Functional properties and performance of the membranes modified with CDs.

Polymer	Modifier	Elements in CDs’ Groups	WCA (Degree)	Filtration					Ref.
				PWP, (L m^−2^h^−1^bar^−1^)	Solution				
					Component	Retention (%)	*FDR* (%)	*FRR* (%)	
PES	-		70	180	BSA	100	77	72	[74]
	CDs	O	53	470		98	54	95	
PS	-	-	87	110	BSA	65	58	58	[179]
	CDs	O, N, S	62	230		85	30	98	
PVDF, PAM/ polyester	CDs	O	-	800	BSA, humic acids, Na alginate, dyes	-	8	-	[178]
	CDs	O, N	48	15		96	≈5	-	
	CDs-βCDx	O	-	115		97	9	-	
	CDs-βCDx	O, N	-	15		98	≈5	-	
	CDs-βCDx	O, N	55	14		96–99	5–62	86–97	
Acetylcellulose	-	-	58	440	BSA, CuSO_4_	99	85	73	[180]
	CDs	O	56	520		98	-	-	
	CDs-amine	O, N	55	580		97	-	-	
	CDs-zwitterions	O, N, S	54	630		>99	90	89	
PVDF	-	-	92	1100	Proteins of milky whey	0	93	-	[70]
	ZrO_2_	-	73	750		82	90	-	
	CDs/ZrO_2_	O, N	76	400		100	82	-	
Polycarbonate	CDs/AgNPs	O, N, S	-	195	oil/water	99	-	-	[181]
PS	-	-	81	24	dye	27	21	90	[182]
	CDs/AgNPs	O	67	65		52	18	84	

**Table 10:** Functional properties and performance of filtration photocatalytic membranes.

Polymer	Modifier	WCA Degree	Substance	Filtration					Ref.
				Scheme */ Light *	Permeability (L m^−2^h^−1^bar^−1^)		Removal (%)	*FRR* (%)	
					Water	Permeate			
PVDF	-	85	COD of dye solution	II/UV	60	48	35	18	[210]
	TiO_2_	45			70	60 _L_	30 _D_, 65 _L_	58 _L_	
ePTFE/grafted PAA	-	85	BSA	II/VIS	50	7	10	-	[69]
	TiO_2_	50			270	90 _L_	90 _L_	10 _D_, 30 _L_	
	TiO_2_/Au	60			260	88 _L_	90 _L_	30 _D_, 95 _L_	
PES	-	72	BSA	II/UV	-	23	95	63	[105]
	TiO_2_	60			-	50 _D_, 150 _L_	96 _D_, 97 _L_	77 _D_, 82 _L_	
	TiO_2_/GO	35			-	115 _D_, 280 _L_	98 _D_, 98 _L_	85 _D_, 92 _L_	
Substrate of RO membrane	-	-	oil refinery wastewater, COD	III/UV	98	75	10	-	[62]
	TiO_2_/GO	-			52	46 _L_	12 _D_ 23 _L_	75 58 **	
PVDF	-	85	river water (COD)	II/UV	-	-	35	55	[211]
	Carbon/Bi_2_O_3_/TiO_2_	45			-	-	70 _D_, 80 _L_	75 _D_, 80 _L_	
PVDF	-	85	PAC	II/VIS	-	22	22	-	[206]
	ZnO/Ag_2_CO_3_/ /Ag_2_O	78			-	33 _D_, 50 _L_	22 _D_, 80 _L_	-	
PVDF	-	-	Oil/water	I-II/VIS	155	20	>97	22	[208]
	TiO_2_	-			-	-		33 _D_, 72 _L_	
	BiVO_4_	-			720	464		66 _D_, 85 _L_	
	WO_3_	-			636	70		40 _D_, 100 _L_	
	TiO_2_/BiVO_4_	-			-	-		52 _D_, 90 _L_	
	TiO_2_/BiVO_4_/ WO_3_	-			-	-		40 _D_, 83 _L_	
PVDF	-	69	Dye	I-I/UV	-	-	-	60	[207]
	WO_3_	-			-	-	-	91 _L_	
	WO_3_/Ti_3_C_2_T_x_	36			-	-	-	93 _L_	
PVDF	-	-	dairy wastewater (COD)	I-II/VIS	-	-	55	25 _L_	[209]
	TiO_2_/BiVO_4_	-			-	-	52	22 _L_	
	TiO_2_/BiVO_4_/ /CNTs	-			-	-	58	20 _L_	
PVDF	Ti_3_C_2_T_x_	54	PACs	II/UV	250	175	3–10 _D_, 25–35 _L_	-	[213]
	TiO_2_/Ti_3_C_2_T_x_	18			280	270	15–30 _D_ 60–90 _L_	75_L_	
Substrate of RO membrane	-	-	dye	IV/UV	150	32	50	-	[205]
	TiO_2_	-			70	32	95 _L_	-	
	TiO_2_/GO	-			30	18	95 _L_	-	

D and L subscripts correspond to the dark and light conditions. * Schemes: I-I. filtration without irradiation → rinsing simultaneously with irradiation; I-II. filtration without irradiation → rinsing followed by irradiation; II. Filtration with irradiation → rinsing with irradiation; III. Filtration with irradiation → rinsing without irradiation; IV. Filtration with irradiation. ** rinsing with HCl.

**Table 11:** Advantages and disadvantages of inorganic modifiers.

Modifier	Advantages	Disadvantages
Phosphates of multivalent elements	Cheap and available reagents, simple synthesis procedure, stability in acidic solutions, no swelling, non-toxicity	Hydrolysis in alkaline solutions (especially for amorphous modifications), high weight of the phosphates of some metals, poor dispersibility in polymer solutions
Oxides of multivalent elements particularly photocatalysts	Cheap and available reagents, simple synthesis procedure, ease of functionalization, photocatalytic activity of some crystalline materials, no swelling, non-toxicity of some oxides, such as TiO_2_, ease of functionalization	Energy consumption for annealing, dissolution in acidic media (slower for crystalline modifications) and in alkaline solutions (Al_2_O_3_), degradation of catalytic activity, high weight, poor dispersibility in polymer solutions
Natural clay minerals	Available and cheap feedstock, cheap reagents and simple procedure for fragmentation, no swelling of zeolites, chemical stability, non-toxicity, ease of functionalization	Swelling of some clay minerals in water, poor compatibility of long rigid nanorods with polymers, poor dispersibility in polymer solutions
Synthetic aluminosilicates	Cheap and available reagents, simple “green” synthesis procedure, no swelling, ease of functionalization, chemical stability, non-toxicity	Energy consumption for hydrothermal synthesis, poor dispersibility in polymer solutions
GO	Cheap and available feedstock and reagents, lightweight, formation of functional groups during synthesis, good dispersibility in water and polymer solutions, chemical stability, possibility of forming a surface layer, ease of functionalization, antibacterial activity	Complex synthesis procedure involving aggressive reagents, poor control of particle size and chemical composition, swelling in water toxicity, dense film causing low fluid permeability, structural defects depressing retention, high cost
rGO	Cheap and available reagents (some are eco-friendly) for GO reduction, chemical stability, low swelling, good dispersibility in polymer solutions, compatibility with polymers, light weight, highly permeable loose layer, ease of functionalization	Expensive feedstock (GO) and reagents for functionalization, toxicity, poor control of particle size and chemical composition, poor dispersibility in water, structural defects from incomplete reduction, high cost
CNTs	Cheap feedstock and reagents for functionalization, compatibility with polymers, lightweight, chemical stability, ease of functionalization, antibacterial activity	Complex and ecologically dangerous synthesis procedure involving high energy consumption, poor control of particle size and chemical composition, poor water dispersibility, aggregation, toxicity, high cost
CDs	Cheap or low-cost feedstock, eco-friendly synthesis procedure, formation of functional groups during synthesis, good dispersibility in water and polymer solutions, lightweight, chemical stability, ease of functionalization, antibacterial activity	Poor control of particle size and chemical composition during synthesis, toxicity, high cost
MXenes	Formation of functional groups during synthesis, good dispersibility in water and polymer solutions, highly permeable layer, ease of functionalization, antibacterial activity	Expensive reagents, complex and ecologically dangerous synthesis procedure requiring high energy consumption, poor control of particle size, chemical degradation, toxicity, high cost
AgNPs	Simple and eco-friendly synthesis procedure, good dispersibility in water and polymer solutions, antibacterial activity	Expensive Ag salt, high weight, poor dispersibility in polymer solutions, chemical degradation, toxicity, high cost

## Data Availability

No new data were created or analyzed in this study. Data sharing is not applicable to this article.

## Abbreviations

AFM	Atomic force microscopy
AgNPs	Silver nanoparticles
BSA	Bovine serum albumin
CDs	Carbon nanodots
CNTs	Carbon nanotubes
COD	Chemical oxygen demand
ePTFE	Expanded polytetrafluorone ethylene
*FDR*	Flux decline ratio, %
*FRR*	Flux recovery ratio, %
g-C_3_N_4_	graphitic carbon nitride
GO	Oxidized graphene
MF	Microfiltration
MWCO	Molecular weight cut-off
NF	Nanofiltration
OVA	Ovalbumin
PAA	Polyacrylic acid
PAC	Pharmaceutically active compounds
PAM	Polyamide
PAN	Polyacrylonitrile
PEI	Polyethyleneimine
PES	Polyethersulfon
PVC	Polyvinyl chloride
PPP	Polypropylene
PS	Polysulfone
PVA	Polyvinylalcohol
PVDF	Polyvinylidene fluoride
PWP	Pure water permeability
PZC	Point of zero charge
rGO	Reduced graphene
RO	Reverse osmosis
ROS	Reactive oxygen species
SEM	Scanning electron microscopy
UF	Ultrafiltration
WCA	Water contact angle, degree
